# CRISPR-C: circularization of genes and chromosome by CRISPR in human cells

**DOI:** 10.1093/nar/gky767

**Published:** 2018-08-24

**Authors:** Henrik Devitt Møller, Lin Lin, Xi Xiang, Trine Skov Petersen, Jinrong Huang, Luhan Yang, Eigil Kjeldsen, Uffe Birk Jensen, Xiuqing Zhang, Xin Liu, Xun Xu, Jian Wang, Huanming Yang, George M Church, Lars Bolund, Birgitte Regenberg, Yonglun Luo

**Affiliations:** 1 Department of Biology, Faculty of Science, University of Copenhagen, Denmark; 2 Department of Biomedicine, Aarhus University, Denmark; 3 BGI Education Center, University of Chinese Academy of Sciences, Shenzhen, China; 4 BGI-Qingdao, Qingdao 266555, China; 5 BGI-Shenzhen, Shenzhen 518083, China; 6 China National GeneBank, BGI-Shenzhen, Shenzhen 518120, China; 7 eGenesis, Inc., Cambridge, MA 02139, USA; 8 Department of Clinical Medicine, Aarhus University, Denmark; 9 James D. Watson Institute of Genome Science, 310008 Hangzhou, China; 10 Department of Genetics, Harvard Medical School, Boston, MA 02115, USA; 11 Lars Bolund Institute of Regenerative Medicine, BGI-Qingdao, Qingdao 266555, China

## Abstract

Extrachromosomal circular DNA (eccDNA) and ring chromosomes are genetic alterations found in humans with genetic disorders. However, there is a lack of genetic engineering tools to recapitulate and study the biogenesis of eccDNAs. Here, we created a dual-fluorescence biosensor cassette, which upon the delivery of pairs of CRISPR/Cas9 guide RNAs, CRISPR-C, allows us to study the biogenesis of a specific fluorophore expressing eccDNA in human cells. We show that CRISPR-C can generate functional eccDNA, using the novel eccDNA biosensor system. We further reveal that CRISPR-C also can generate eccDNAs from intergenic and genic loci in human embryonic kidney 293T cells and human mammary fibroblasts. EccDNAs mainly forms by end-joining mediated DNA-repair and we show that CRISPR-C is able to generate endogenous eccDNAs in sizes from a few hundred base pairs and ranging up to 207 kb. Even a 47.4 megabase-sized ring chromosome 18 can be created by CRISPR-C. Our study creates a new territory for CRISPR gene editing and highlights CRISPR-C as a useful tool for studying the cellular impact, persistence and function of eccDNAs.

## INTRODUCTION

The Clustered Regularly Interspaced Short Palindromic Repeats (CRISPR) and CRISPR-associated protein 9 (Cas9) is an adaptive immune system in bacteria and archaea that eliminate phages (1). Mediated by a small guide RNA (gRNA), the endonuclease Cas9 is harnessed for gene editing and has quickly become a highly attractive and powerful tool in basic and applied research (2–4). The fidelity and nuclease-precision of the CRISPR–Cas9 system insures no or minimal off-target effects and facilitates fast, functional gene studies of e.g. site-directed mutations in eukaryotic cells. Moreover, CRISPR–Cas9 mediated gene knockouts can be created through a pair of gRNAs that triggers dual double-stranded DNA breaks at essential exons, leading to DNA deletion and functional disruption of the gene encoded protein (3,4) or the inactivation of porcine endogenous retroviruses (5).

However, it is commonly thought that excised DNA after dual restriction by CRISPR–Cas9 will degrade or dilute out through cell proliferations, yet the fate of deleted chromosomal DNA is still unknown. Any nuclear double-stranded DNA breakage will in principle be recognized by the DNA repair machinery and two breaks on the same chromosome could led to mistakes, either by end joining or by homology-directed repair of the deleted linear DNA fragment, resulting in circular DNA formation. DNA deletions and DNA inversions have previously been shown to form after dual restriction by CRISPR–Cas9 (6,7) but it remains to be resolved whether deleted DNA fragments also form extrachromosomal circular DNA (eccDNA). Genome-scale studies have recently shown that eccDNAs are common elements in human cells (8,9) and malignant tumors often carry oncogene amplifications on eccDNAs, known as double minutes (10–13). Chromosome-derived eccDNAs appear to arise mostly at random, although certain eccDNA types form more frequently (8,9,14). Despite mounting evidence of eccDNA prevalence, a direct connection between DNA deletions and eccDNA formation has so far only been found sporadically (8,12,15,16) and for fifty years, cancer studies of double minutes (microscopically visible eccDNAs) has been prohibited by limited cell models and eccDNA detection techniques, underlying the need for more directed genetic tools to facilitate eccDNA analyses.

In this study, we first created an exogenous dual-fluorescence biosensor cassette (ECC biosensor) for studying the biogenesis of a fluorophore gene-encoded eccDNA in human cells. We discovered that a targeted DNA deletion in the ECC biosensor, by a pair of CRISPR/Cas9 gRNAs, can form green fluorescent protein expressing eccDNA in human cells after end-to-end DNA ligation. This technology of generating extrachromosomal circular DNA by CRISPR was hereafter referred as CRISPR-C. Using CRISPR-C, we further demonstrated generation of eccDNA from intergenic and genic loci and confirmed formation of eccDNAs in human cells, in sizes from a few hundred base pairs up to a 47.4 megabase-sized ring chromosome, chr18.

## MATERIALS AND METHODS

### Oligonucleotides

All oligonucleotides are ordered from Merck KGaA, Darmstadt, Germany. The sequences for all oligonucleotides used can be found in Supplementary Table S1 and S2.

### Cell culture

Tissue cultures of human embryonic kidney 239T (HEK293T) cells and normal immortalized human fibroblasts (HMF, clone MJ2646, a gift from Dr Bin Liu, Danish Cancer Society, Denmark) were cultured in Dulbecco's modified Eagle's medium (DMEM) (LONZA) supplemented with 10% fetal bovine serum (FBS) (Gibco), 1% GlutaMAX (Gibco), and penicillin/streptomycin (100 units penicillin and 0.1 mg streptomycin/ml) in a 37°C incubator with 5% CO_2_ atmosphere and maximum humidity. HEK293T and MJ2646 cells were passaged typically every 2–3 days at 1:8 and 1:3 ratio, respectively, when reaching approximately 90% confluence. This was done by washing cells twice (equal volume as growth medium) gently with phosphate buffered saline (PBS, 1×) without calcium and magnesium, followed by cell detachment by 0.05% Trypsin-EDTA for 3–5 min at 37°C.

### Resource of plasmids

The following vectors were used: lentiCRISPRv2 (a gift from Feng Zhang, Addgene plasmid # 52961) and pUC19 (gift from Joachim Messing, Addgene plasmid # 50005). The pMAX-GFP (VDF-1012) was supplied by the Amaxa nucleofection kit. The PiggyBac hybase plasmid is generously provided by Prof. Jacob Giehm Mikkelsen from the Department of Biomedicine, Aarhus University (http://www.giehmlab.dk). Alternatively, the PiggyBac transposase expression vector can be purchased from SBI system Biosciences (Cat. # PB210PA-1).

### Generation of eccDNA biosensors

The ECC biosensor fragments were synthesized by Gene Universal Inc. (229A Lake Dr Newark De 19702, USA) and validated by Sanger sequencing. The ECC biosensor fragments, which contains fluorophore gene markers of enhanced green fluorescent protein (EGFP) and the monomeric derivative of DsRed fluorescent protein (mCherry), were sub-cloned into a PiggyBac transposon vector (from Prof. George M. Church's group, http://arep.med.harvard.edu) carrying the *pac* gene, encoding a puromycin N-acetyl-transferase that confer resistance to puromycin (see Supplementary Figures S1 and S2). The final PiggyBac transposon-based ECC biosensor plasmids (CAG-ECC and TRE-ECC) were verified by restriction enzyme digestion.

### Generation of CRISPR/Cas9 gRNAs

All CRISPR gRNAs were designed with the online software tool CRISPOR v4.2. The gRNAs were selected selecting based on (i) minimal off-targets, i.e. off-targets that typically requires more than three mismatches for perfect annealing elsewhere; (ii) predicted activity over 30% provided by the CRISPOR web tool; (iii) avoid strong secondary structure of the guide sequences and (iv) absent of poly-thymine (max 3) and the BsmBI restriction site in the guide. All CRISPR gRNA oligos with the corresponding overhangs used for Golden Gate Assembly were ordered from Merck KGaA, Darmstadt, Germany (Supplementary Table S1).

To generate CRISPR gRNA expression vector, we used a previous optimized protocol. Briefly, for each gRNA, two complementary guide oligonucleotides (100 pmol each) in 1× NEB buffer 2 (in a total volume of 20 μl) were first denatured at 95°C for 5 min, using a heating block, followed by slow annealing by turning off the heating block. For Golden Gate Assembly, the following reaction mix was prepared: 1 μl of the above annealed oligonucleotides, 100 ng of the lentiCRISPRv2 plasmid, 1 μl FastDigest BsmBI restriction enzyme (Thermo Fisher Scientific), 1 μl T4 Fast ligase (Thermo Scientific), and 2 μl T4 ligase buffer in a total volume of 20 μl. Golden Gate Assembly was performed in a thermal cycler using the following program: Ten cycles of 37°C for 5 min and 22°C for 10 min; one cycle of 37°C for 30 min; and one cycle of 75°C for 15 min. The ligation product was stored at 4°C or used directly (2 μl ligation product) to transform competent *Escherichia coli* cells. Using this protocol, we have experienced that over 95% of the bacterial clones are positive by PCR screening using a U6 forward primer (5′-GAGGGCCTATTTCCCATGATTC-3′) and the antisense guide oligonucleotide (template strand of the gRNA spacer). Sanger sequencing validated all gRNA vectors used in this study.

### Cell transfection

All transfections in this study were conducted with the X-tremeGENE 9 transfection reagent (Roche) in 24-well plate, if not stated elsewhere. Briefly, 60,000 cells, counted with nucleocounter NC-100 (Chemometec), were typically seeded into a 24-well plate one day before transfection. For each transfection, a total of 250 ng plasmid DNA and 0.75 μl X-tremeGENE 9 transfection reagent (1:3 ratio of μg total DNA relative to transfection reagent), were mixed in 50 μl OptiMEM (Gibco) and incubated at room temperature for 15 min. The transfection mixture was homogeneously added to the cells. Unless stated elsewhere, for transfection of pairs of CRISPR plasmid, equal amount of each CRISPR plasmid was used. As transfection control, pUC19 or GFP plasmid was used for control groups. For evaluation of endogenous eccDNA formation by CRISPR pairs, 1.5 × 10^6^ HEK293T cells or 2.2 × 10^6^ HMF cells were seeded in 10-cm cell culture dish and transfected next day at ∼50% cell confluence.

### Generation of ECC biosensor stable clones

Stable ECC biosensor clones were generated by transfection of 60,000 HEK293T cells with 200 ng ECC biosensor plasmid and 50 ng PiggyBac hybase plasmid using X-tremeGENE 9. Twenty four hours post transfection, transfected cells were cultured in complete medium supplemented with puromycin (1 μg/ml). One-week after transfection, the puromycin resistant cells were trypsinized, single cell was manually picked under a stereomicroscope and cultured in 96-well until 70–80% confluence. Single clones normally evolve after 14 days in culture. The stable clones were further expanded and the copy number of the integrated ECC biosensor was assessed by Southern blot, probing for *EGFP*, using the ECC biosensor plasmid as reference in 1, 5 and 10 copies/genome.

### Fluorescence-activated cell sorting (FACS)

Cells, dissociated with 100 μl trypsin-EDTA, were suspended in 100 μl 5% FBS–PBS and transferred to a 96 deep-well plate on ice. Cells were spun down ∼800 × g (2000 rpm) for 5 min and the supernatant was removed by gently inverting the plate. For fixation, 200 μl 4% paraformaldehyde was added to the cells and mixed gently by pipetting. After fixing for 15 min at room temperature, the fixed cells were washed twice with 5% FBS–PBS. Lastly, cell pellets were re-suspended in 500 μl PBS and cells were kept at 4°C until FACS analysis. FACS was performed using a BD LSRFortessa (supported by the FACS CORE facility, Department of Biomedicine, Aarhus University) with at least 30 000 events collected for each sample. Data were analyzed using Flowjo software.

### Time-course analysis of ECC biosensor

The CAG-ECC (clone #1–9) and TRE-ECC (clone #1–15) were transfected with Cr1 and Cr2. 72 hours after transfection (defined as passage 1), the transfected cells were dissociated with 100 μl trypsin-EDTA (37°C for 5 min) and re-suspended in 200 μl 5% FBS-PBS medium. One-third of the cells were analyzed by FACS, one-third of the cells were used for PCR analysis, and the remaining one-third of the cells were passaged to a new 24-well plate. The cells were passaged every second day at a one-third ratio until passage 6.

### EccDNA purification in human cells

Cells were harvested typically at 48 h after transfection or harvested from a fraction of the transfected cells in time-course experiments at various passages. Suspended, trypsinized cells were pelleted at 425 x *g* (2000 rpm), 5 min. The supernatant was removed and cells were lysed for eccDNA analysis (see the following optimization method); either by genotyping, using outward PCR and Sanger sequencing, or by Southern blot.

### Optimization of endogenous eccDNA purification

To assess the methods for endogenous eccDNA purification, three different protocols were tested before outward PCR: (i) genomic DNA column purification; (ii) cell lysate; (iii) plasmid DNA column purification. For each of the three methods, cells were counted and fractionated in three different concentrations: 10^5^ cells, 10^4^ cells and 10^2^ cells. Then, to each cell sample was spiked in 10^5^ copies of pUC19 plasmid as internal control and protocols (i); (ii) or (iii) were executed.

#### Genomic DNA column purification

Genomic DNA was purified according to protocol (#056-60, A&A Biotechnology). In brief, cells were lysed by protease for 30 min at 55°C, the RNA was removed by RNase A/T1 mix (2 μg/sample, Thermo) and genomic DNA was washed and purified by gravity flow on an ion exchange membrane (AX tissue), followed by DNA precipitating at 14,000 x g for 30 min at 2°C, 70% ethanol wash and finally dissolving the DNA pellet in 40 μl Tris–Cl 10 mM, pH 8.0. Of the total 40 μl DNA solution, 32 μl was transferred for linear DNA removal, saving the remaining 8 μl at −20°C until PCR analysis.

#### Cell lysate

Cells were suspended in 0.2 ml lysis buffer (KCl 50 mM, MgCl_2_ 1.5 mM, 0.5% NP40 and 0.5% Tween 20, 10 mM Tris pH 8.5) and incubated with 10 μl proteinase K (19.1 mg/ml, Thermo) for 2.5 h at 55°C. Proteinase K was heat-inactivated at 95°C for 10 min, cooled down at room temperature for 15 min, and 32 μl DNA solution was transferred for linear DNA removal, storing the rest of the cell lysate at –20°C until PCR analysis.

#### Plasmid DNA column purification

Plasmid DNA from cells was purified according to protocol (#010-50, A&A Biotechnology) after completed cell lysis in 0.6 ml L1 suspension buffer with 30 μl proteinase K (19.1 mg/ml, Thermo) for 6 h at 55°C, and removal of RNA by RNase A/T1 mix (2 μg/sample, Thermo) at room temperature for 10 min. Plasmid DNA was washed and purified by gravity flow on an ion exchange membrane (Plasmid 10 AX), followed by DNA precipitating at 14 000 x g for 30 min at 2°C, 70% ethanol wash and finally dissolving the DNA pellet in 40 μl Tris–Cl 10 mM, pH 8.0. Of the total 40 μl DNA solution, 32 μl was continued for linear DNA removal, saving the remaining DNA at –20°C until PCR analysis.

### Removal of linear DNA

A maximum of 4–5 μg total DNA (most cases < 2 μg) was digested with four fast digest units (Thermo Scientific), using an endonuclease that did not cleave investigated eccDNA, e.g. for most experiments *XbaI* (endogenous eccDNA) or *EcoRI* (ECC biosensor) was used. Endonuclease digestion was completed after 1 hour in a 40 μl volume at 37°C, followed by heat-inactivation, according to enzyme protocol (e.g. 65°C, 20 min). To the total 40 μl endonuclease digested DNA was added a 35 μl Plasmid-Safe DNase mixture (Epicentre, Illumina), containing 1.5 μl Plasmid-Safe DNase (15 units), 3 μl ATP (25 mM), 7.5 μl 10× DNase reaction buffer and 23 μl H_2_O. Samples were incubated in a heating block at 37°C for 16 h. Next day, linear DNA digestion was continued by adding 5 μl fresh Plasmid-Safe DNase mixture every two hours, consisting of 1.5 μl Plasmid-Safe DNase (15 units), 3 μl ATP (25 mM ATP) and 0.5 μl 10× DNase reaction buffer and when reaching 90 units and ∼24 h digestion, DNase was heat-inactivated at 70°C for 30 min (see Supplementary Figures S13 and S14). In case of the cell lysate protocol, the quick protocol included max 4 μg DNA, endonuclease digestion of a 40 μl cell lysate volume, conducted directly after cell lysis in 1× fast digest buffer at 37°C for 16 h, in combination with 10 units Plasmid-Safe DNase and 2 μl ATP (25 mM). After heat-inactivation of the endonuclease, the Plasmid-Safe DNase treatment was continued for additional 24–48 h (dependent on DNA input), adding fresh Plasmid-Safe DNase mixture step-wise and reaching up to 100 units/sample.

### Polymerase chain reaction (PCR)

All PCR reactions were performed with the high-fidelity platinum *Pfx* polymerase in the presence of 2× enhancer solution (#11708013, Thermo). All PCR primers were ordered from from Merck KGaA, Darmstadt, Germany (Supplementary Table S2).

### Southern blot analysis

Southern blot was performed using an optimized method from our group. In brief, a total 15–25 μg genomic DNA was digested with *EcoRI* restriction enzyme overnight. The enzyme digested DNA was separated on 1% agarose gel for 16 h at 30 V. The DNA was transferred on to a membrane by vacuum blotting. PCR primers for generating the probe: 5′-ATTCACCGTCTCATCCAGCGAGGGCGATGCCACCTAC-3′ and 5′-TAAGTCGGTCTCATCAGTGGTTGTCGGGCAGCAGCAC-3′ (500 bp EGFP probe). Probe labelling was performed using the Prime-It II Random Primer Labeling Kit according to the manufacturer's instructions. Pre-hybridization and hybridization steps were carried out at 42°C. Excess probe was washed from the membrane with SSC buffer, and the hybridization pattern was visualized on X-ray film by autoradiography. A detailed protocol with step-by-step guidance can be found at our group page: www.dream.au.dk.

### Karyotyping

Cells were incubated with Chromosome Resolution Additive, diluted 1:10 000, and 500 ng/ml cholcemide for 2.5 h at 37°C. The culture was harvested by incubating with TrypLE for 10–20 min at 37°C. Cells were collected by centrifugation and the pellet was re-suspended in 0.4% KCl, gently vortexed and subsequently incubated for 30 min at 37°C. After re-centrifugation, the pellet was re-suspended by adding fixative drop-wise up to 2 ml using 1 volume acidic acid and 3 volume methanol. After 20 min fixation at room temperature, cells were spun down and the fixation step was repeated twice. Chromosome spreads were prepared by dripping the cell-suspension onto wet glass slides in a humid atmosphere at 37°C. The slide glasses were stained, using quinacrine and mounted in antifade solution.

### Fluorescent in situ hybridization (FISH) of telomere and whole chromosome 18 painting

Slides were made with each of the cells suspensions prepared for Karyotyping. Hybridization with PNA-telomere probe in red was performed, followed by DAPI as counterstain. Automated scanning of the slides was applied to identify and locate metaphases, and image identified metaphases were captured using the automated scanning software. The telomere probe was stripped and re-hybridization with whole chromosome 18 probes in green followed by DAPI as counterstain. Relocation of previously captured metaphases followed by another capture of painted metaphases was carried out. For wildtype parental cells, we analyzed 65 metaphases. For CRISPR transfected cells, we analyzed 113 metaphases. A more detailed FISH protocol can be acquired from the authors.

### Sanger sequencing

All Sanger sequencing in this study were conducted using the Mix2Seq Kit in Eurofins Genomics (Munich, Germany). Sanger sequencing results were analyzed with SnapGene Viewer or four peaks software.

### Deep sequencing

Purified PCR products were mixed with End Repair Mix (BGI Tech.), incubated at 20°C for 30 min, then purified with QIAquick PCR purification kit (Cat No./ID: 28104). Purified PCR products were mixed with an A-Tailing reaction (BGI Tech.), incubated at 37°C for 30 min and purified with QIAquick PCR purification kit. PCR-free adaptor ligation was carried out by mixing the above 3′ adenylate DNA, PCR Free index adapters (BGI Tech.), T4 DNA Ligase and buffer. The reaction was incubated at 16°C for 16 h and purified with QIAquick PCR purification kit. The ligated PCR products were purified by gel-electrophoresis (2% agarose) and the QIAquick Gel Extraction Kit (Qiagen). The molecular length of the final sequencing library was determined with Agilent 2100 bioanalyzer (Agilent DNA 1000 Reagents) and sequenced with HiSeq2500 System (HiSeq SBS Kit V4, Illumina).

### Analysis of amplicon sequencing results

The theoretical circular sequences of [*dsCRP^circle 1q23.2:0.57 kb^*] and [*TRIM28^circle exon 1-2^*] were generated based on human hg38. Clean reads were aligned against the theoretical reference using the BWA-mem algorithm. Duplicate removing was not suitable for amplicon sequencing and thus omitted. Reads across the theoretical end joining site (junction) were used for indel-calling. Soft-clip reads were omitted.

### Statistics

Analysis of correlation was conducted with Pearson rank test.

## RESULTS

### Generation of an ECC biosensor and circular DNA induction by CRISPR-C

**Figure 1.**
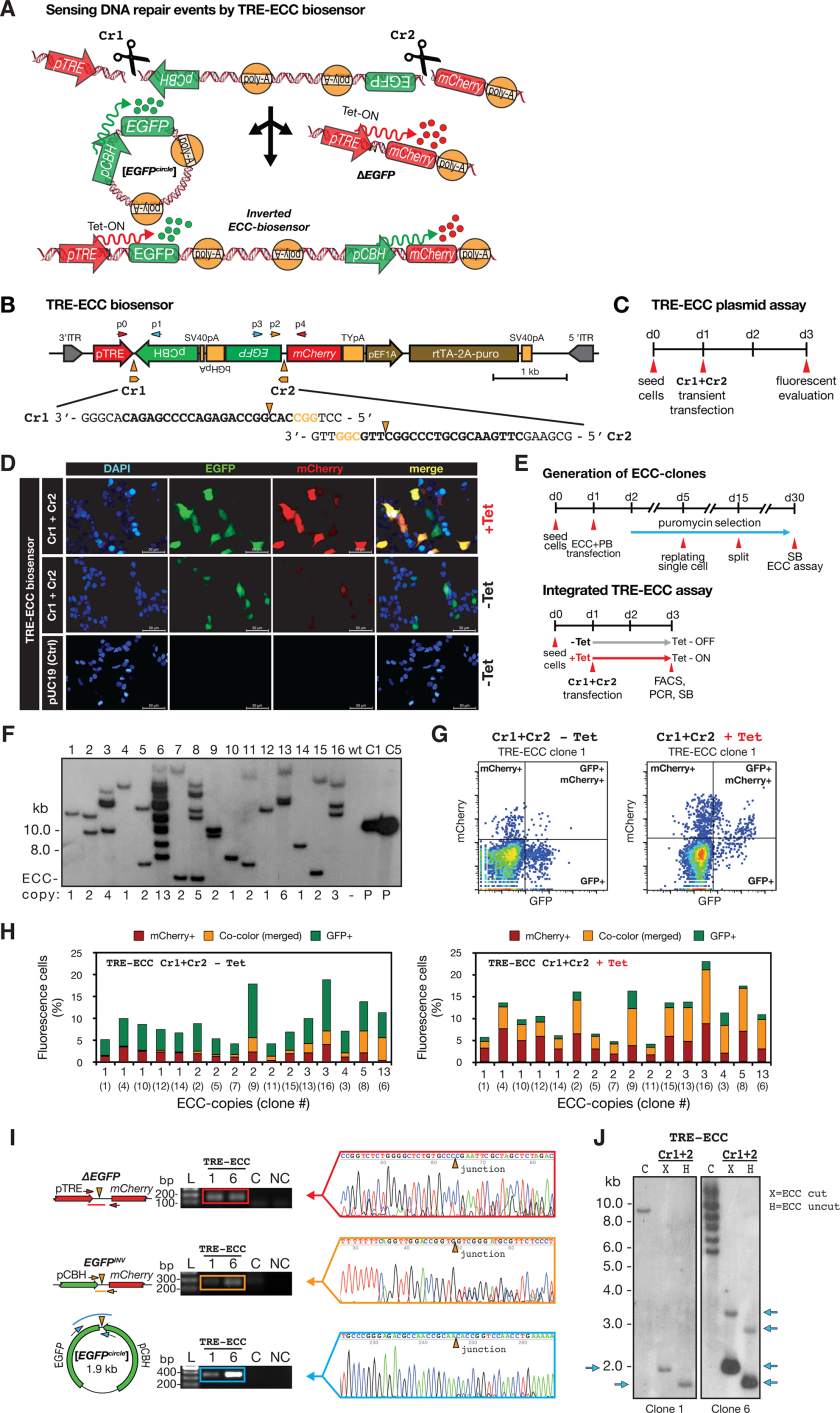
A dual-fluorescence eccDNA biosensor system and CRISPR-C for studying eccDNA biogenesis in human cells. (**A**) Model of TRE-ECC biosensor after two double-stranded breaks by CRISPR-C (Cr1 and Cr2), leading to [*EGFP^circle^*] and Δ*EGFP* or *EGFP* inversion. (**B**) Scheme of TRE-ECC biosensor. (**C**) Experimental outline. (**D**) TRE-ECC plasmid assessment by fluorescence microscopy after Cr1+Cr2 using pUC19 as control (uncut). (**E**, upper part) Outline of stable genomic integration of TRE-ECC in HEK293T. (**F**) Copy-number assessment by Southern blot with EGFP-probe after *KpnI* digestion. (E, lower part) Outline for Cr1+Cr2 activation of integrated TRE-ECC. (**G**) Representative result from FACS analysis of clone 1 in the absence or presence of tetracycline (Tet). (**H**) Histograms of fluorescence cell percentages of all 15 isolated TRE-ECC clones after Cr1+Cr2 and FACS analysis. (I) PCR and Sanger sequencing validation of genotypes on exonuclease-treated DNA, displayed in A, after Cr1+Cr2 for TRE-ECC clone 1 and clone 4. C, negative CRISPR control; NC, non-template control. (**J**) Southern blot, probed with EGFP on HEK293T purified and digested DNA from untreated (C, *KpnI*) and Cr1+Cr2 treated cells. X = *XbaI*, H = *HindIII*.

To enable real-time monitoring of eccDNA biogenesis in cells, we designed and synthesized a dual-fluorescence biosensor cassette (ECC) with constitutive gene-expression: CAG-ECC (Supplementary Figure S1) as well as a tetracycline inducible biosensor: TRE-ECC (Figure 1 and Supplementary Figure S2). The principle for each designed ECC-cassette was to mark the outcome after DNA repair of two introduced site-directed double-strand DNA breaks in cultured human cells, using two CRISPR gRNAs (Cr1 + Cr2) (Figure 1A and Supplementary Figure S3). A genomic DNA deletion, of the sequence encoding enhanced green fluorescent protein (*ΔEGFP*), would lead to functional *mCherry* expression after end-joining with the *mCherry* promoter (pCAG or pTRE). Likewise, *EGFP* expression was expected to mark circularization of the deleted DNA fragment, leading to an [*EGFP^circle^*]. Alternatively, an inversion could also lead to expression of both *EGFP* and *mCherry* but under a different expression scheme (Figure 1A and Supplementary Figure S3).

To experimentally test the ECC biosensor on a plasmid, we transiently transfected human embryonic kidney 293T cells (HEK293T) with CAG-ECC and Cr1+Cr2, along with serial control transfections (Supplementary Figures S4 and S5). As expected, only cells transfected with CAG-ECC and Cr1+Cr2 expressed both *EGFP* and *mCherry*. However, as transient transfection of the CAG-ECC vector could not distinguish eccDNA from inversion events (Supplementary Figure S3) we also tested the inducible TRE-ECC in HEK293T. Here the majority of the cells, in absence of tetracycline, were EGFP positive, indicating [*EGFP^circles^*], while substantially less cells were mCherry positive (Figure 1B–D, Supplementary Figures S6 and S7). Addition of tetracycline to cells enhanced both *EGFP* and *mCherry* expression, supporting formation of [*EGFP^circles^*] as well as EGFP deletion (*ΔEGFP*) and *EGFP* inversions (*EGFP^inv^*) when targeted by CRISPR pairs, hereafter referred as CRISPR-C.

Transient transfection is known to overload cells with many copies of plasmids. To overcome plasmid overloading and to distinguish inversions from eccDNAs, we established stable HEK293T biosensor reporter cells by the PiggyBac transposon system, which recognizes transposon-specific inverted terminal repeat sequences (ITRs) of the transposon vector and efficiently integrates ITRs and the ITR-flanked genes into TTAA chromosomal sites. Nine stable CAG-ECC clones and fifteen stable TRE-ECC clones were established. Each clone carried from one up to thirteen copies of the biosensor cassette, assessed by Southern blotting (Figure 1E–G, Supplementary Figures S8 and S9). Fluorescence-activated cell sorting (FACS) analysis of all individual cell clones, induced by CRISPR-C (Cr1+Cr2), revealed correlation between the ECC biosensor copy number per cell and the median levels of EGFP+/mCherry+ co-colored cells (*R* = [0.79 to 0.93], Pearson rank test, Supplementary Figure S10). It further supported formation of fluorophore transcripts from eccDNA after CRISPR-C (Figure 1H, Supplementary Figures S8 and S9). Topological confirmation of the 1.9 kb-sized [*EGFP^circle^*], along with *ΔEGFP* and *EGFP^inv^*, was obtained by Sanger sequencing of PCR products and [*EGFP^circles^*] were further confirmed by Southern blot analysis for both the TRE- and the CAG-ECC isolated clones (Figure 1I–J and Figure S11).

### CRISPR-C can generate endogenous genic and intergenic eccDNAs in human cells

**Figure 2.**
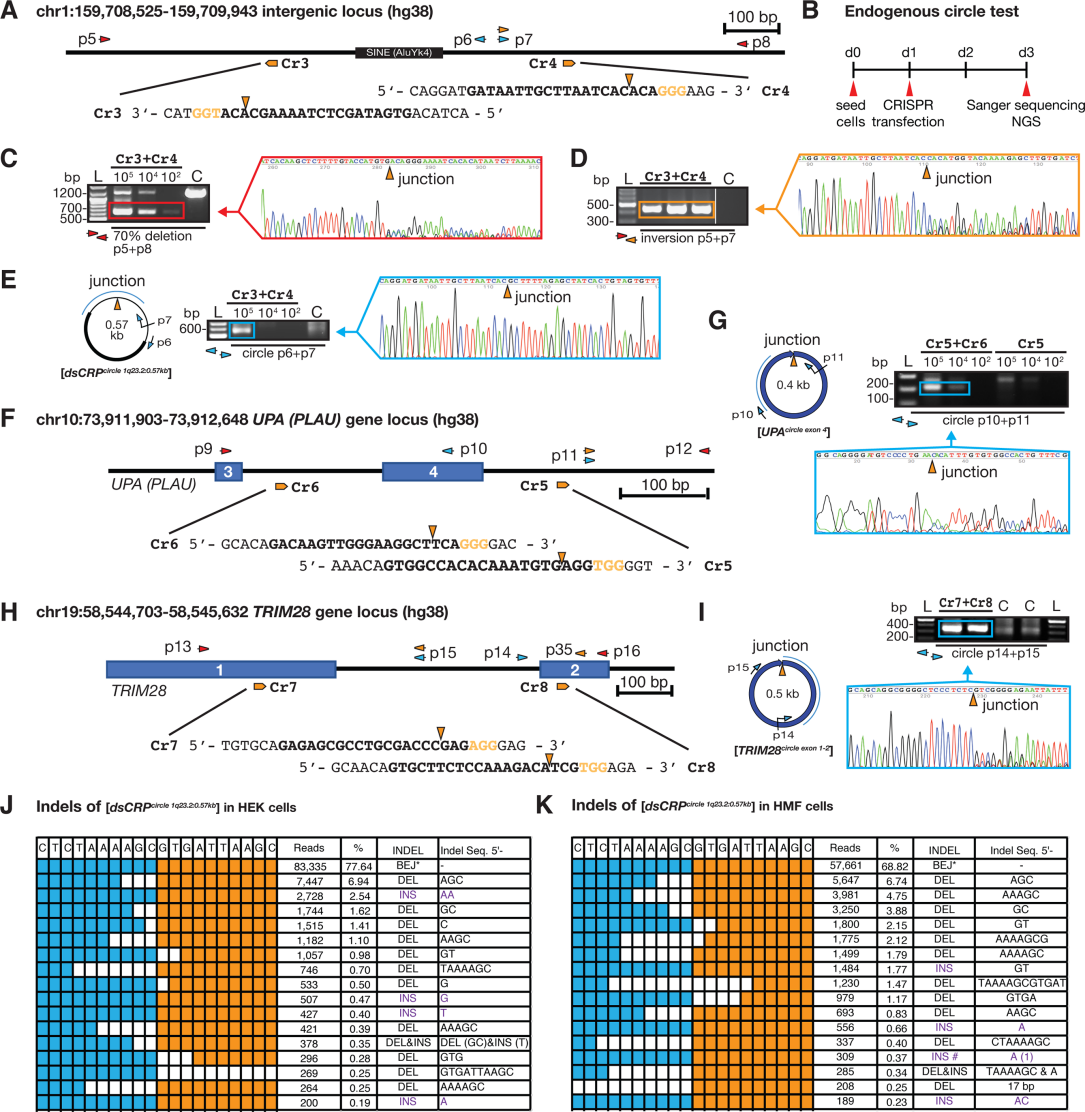
Endogenous DNA circularization by CRISPR-C. (**A**) Chromosome 1 map 2 kb downstream of the *CRP* locus. gRNA: Cr3, Cr4; diagnostic oligos: p5 to p8. (**B**) Experimental outline. (C–E) Gel-images of corresponding PCR products across junctions after testing for (**C**) DNA deletion, 0.64 kb; wildtype, 1.23 kb), (**D**) inversion and (**E**) circular DNA, next to resultant chromatograms from Sanger sequencing. (**F**) Chromosome 10 map at the *PLAU (UPA)* locus. gRNA, Cr5 and Cr6, diagnostic oligos; p9 to p12. (**G**) PCR and sequencing confirmation of the [*UPA^circle exon 4^*]. (**H**) Chromosome 19 map at the *TRIM28* locus. (**I**) PCR and sequencing confirmation of the [*TRIM28^circle exon 1-2^*]. (**J**–**K**) Indel distribution across the junction of [*dsCRP^circle^*] generated in HEK293T and HMF cells, respectively. For (E), (G) and (I), exonuclease-treated DNA was used as template.

Endogenous eccDNA has previously been found in both tumor and normal cells (8,10). We next investigated whether CRISPR-C also could generate eccDNAs in human embryonic kidney cells (HEK293T) and immortalized human mammary fibroblasts (HMF). EccDNAs are commonly derived from intergenic regions and often consist of repeated sequences (8,17). Thus, we designed two CRISPR gRNAs (Cr3+Cr4) targeting a site on chromosome 1 (chr1:159 708 525–159 709 943), which is located 2 kb downstream of the C-reactive protein encoding gene, *CRP*, and contains a SINE (AluYk4) element (Figure 2A). We first estimated the deletion efficiency to ∼50–70% after CRISPR-C (Cr3+Cr4) in HEK293T cells (Figure 2B and C). We confirmed CRISPR inversion events in these cells by PCR and Sanger sequencing (Figure 2D). Finally, and as anticipated, CRISPR-C led to circularization of the deleted DNA, resulting in formation of the 0.57 kb-sized [*dsCRP^circle 1q23.2:0.57 kb^*] (Figure 2E) validated by outward PCR and Sanger sequencing. *CRP* circularization by CRISPR-C was further validated by rolling circle amplification (data not shown). Since HEK293T cells are polyploidy and a CRISPR duplication event potentially could give a false-positive PCR signal for eccDNA (Supplementary Figure S12), we tested and validated three protocols (genomic DNA preparation, cell lysate, and plasmid preparation) for eccDNA purification and exonuclease treatment, in order to remove all linear/chromosomal DNA (Supplementary Figure S13). We found that cost-effective and conventional cell lysate protocol allowed us to detect the [*dsCRP^circle 1q23.2:0.57 kb^*] in 10∧5 cells, using outward directing oligos PCR and Sanger sequencing. Controls confirmed complete removal of linear DNA but not plasmid DNA (Supplementary Figure S13). The eccDNA purification protocols were repeated in HMF cells, reaching comparable results and conclusions (Supplementary Figure S14). Thus, the cell lysate method (∼10^5^ cells and exonuclease treatment) was hereafter used for all proceeding experiments. Taken together, we found that CRISPR-C can generate intergenic eccDNA, both in immortalised human fibroblasts and in human cancer-like cells (HEK293T).

Next, we tested induction of DNA circularization by CRISPR-C at two different genic loci (*urokinase, UPA*; and tripartite motif containing 28, *TRIM28*) as endogenous eccDNAs also are reported to derive from genic sequences (8,10,13,18,19). In line with findings of the [*dsCRP^circle 1q23.2:0.57 kb^*], CRISPR-C led to generation of a [*UPA^circle exon 4^*] and a [*TRIM28^circle exon 1-2^*] both in HMF as well as in HEK293T cells and their structures were confirmed by Sanger sequencing of outward PCR products (Figure 2F–I and Supplementary Figures S15 and S16). Hence, endogenous eccDNA formation by CRISPR-C seems to be a general outcome in cells, since it is cell-line independent and eccDNAs are formed both at genic loci and at intergenic regions.

### CRISPR-C eccDNA mainly forms by end-joining

Sanger sequencing of the circular junctions of [*EGFP^circle^*], [*dsCRP^circle 1q23.2:0.57 kb^*], [*UPA^circle exon 4^*] and [*TRIM28^circle exon 1-2^*] gave evidence of end-joining events. This data support previous notions that the major DNA-repair pathway that appears to mediate eccDNA formation in mammalian cells is non-homologous end joining (NHEJ) (17,18). To better understand the indel events, we further sequenced the DNA across the junction of the [*dsCRP^circle 1q23.2:0.57 kb^*] and the [*TRIM28^circle exon 1-2^*] by next-generation sequencing. For CRISPR-C introduced *dsCRP* eccDNA, we observed 68.8% and 77.6% blunt end-joining (BEJ) in HEK293T and HMF cells, respectively while the remaining 31.2% and 22.4% had a variable indel size up to maximum 17 bp (Figure 2J and K). Similarly, the majority of [*TRIM28^circle exon 1-2^*] indels was less than 4 bp (79%) (Supplementary Figure S16). Though, for [*TRIM28^circle exon 1-2^*], we only observed 6.8% formed by BEJ while a majority (61%) had either one nucleotide insertion (43.3%) or deletion (13.2%) (Supplementary Figure S17). Such an indel distribution has previously been reported after CRISPR-editing (19,20). In sum, our results suggest that the majority of eccDNAs generated by CRISPR-C is formed by an end-joining mediated DNA repair pathway.

### The stability of CRISPR-C generated [*EGFP^circles^*] in human cells

**Figure 3.**
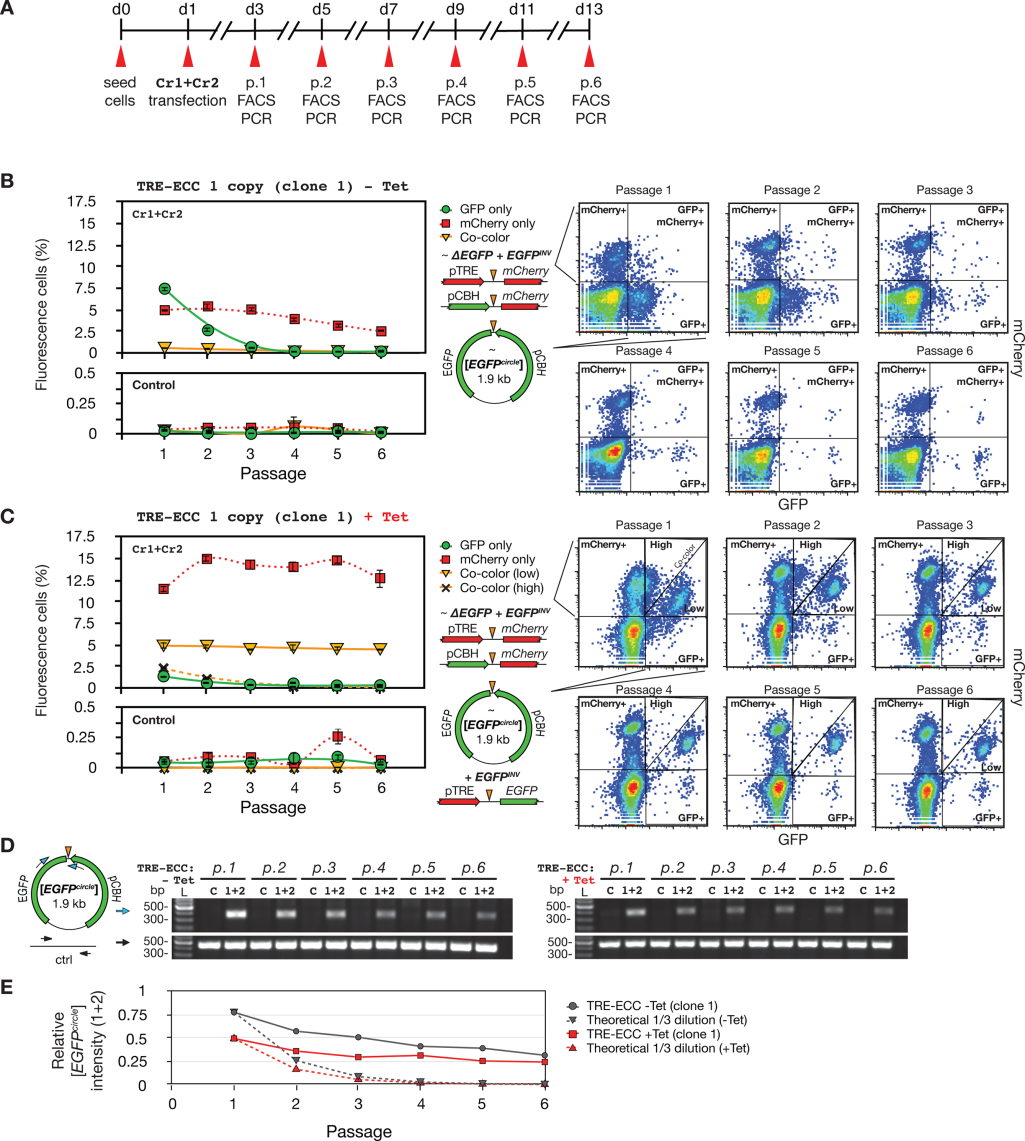
Stability of eccDNAs generated by CRISPR-C. (**A**) Outline of time-course experiment. (**B**) Left, percent fluorescence cells from 1 to 6 cell passages (1:3 split-ratio) in absence of tetracycline (-Tet); right, corresponding FACS gating images. (**C**) As B description, cells propagated in +Tet conditions. (**D**) Outward PCR analysis of [*EGFP^circles^*] at passage 1 to 6; left, -Tet; right, +Tet. Cr1+Cr2 (1+2), CRISPR gRNAs; C, control – gRNA; ctrl, DNA template control. (E) Quantification of PCR analysis by Image J, solid lines; theoretical 1/3 dilution rate, dash lines.

To evaluate the stability of *de novo* generated eccDNA by CRISPR-C, succeeding experiments were carried out using the transgenic TRE-ECC (clone 1) and CAG-ECC (clone 2) with a single copy insertion as an example. A time-course experiment was carried out as shown in Figure 3A and Supplementary Figure S18A for two weeks. One third of the cells were harvested at 6 constitutive passages and analyzed by FACS and outward PCR to detect [*EGFP^circles^*]. At passage 1, the [*EGFP^circle^*] genotype in TRE-ECC cells (-Tet) was mainly found in GFP positive only (GFP+) cells (Supplementary Figure S19). *ΔEGFP* and *EGFP^inv^*were mainly found in mCherry positive only (mCherry+) cells (Supplementary Figure S19). Over the FACS time-course the percentage of GFP+ cells declined rapidly from 7.4% (p.1) to 0.2% (p.4) (Figure 3B). However, the percentage of mCherry+ cells and co-colored (GFP+mCherry+) cells were observed at nearly steady levels from p.1-p.6 (Figure 3B). Likewise, in the TRE-ECC (+Tet) cells, the percentage of GFP+ cells and high co-colored cells (mCherry^high+^, GFP^low+^), which harbored [*EGFP^circles^*] (Supplementary Figure S19), declined rapidly from p.1-p.4, close to the dilution ratio (i.e. 2.7 ± 1.9). Similar to the experiment -Tet (Figure 3B), the mCherry+ cells and low co-colored cells (mCherry^low+^, GFP^high+^) were detected at nearly stable levels (Figure 3C, +Tet). Moreover, comparable time-courses were observed in CAG-ECC experiments (Supplementary Figure S18B and C). Despite the indicative data from FACS, supporting a rapid decrease of cells expressing [*EGFP^circles^*] over time, when we tested for the stability and presence of [*EGFP^circles^*] DNA up to day 13 (six passages) by outward PCR, we found DNA products in all passages. Remarkably, the detected apparent decrease in [*EGFP^circles^*] from PCR analysis was slower than the theoretical dilution rate (Figure 3D and E and Supplementary Figure S18D and E). The PCR results suggest that [*EGFP^circle^*] DNA can stay rather stably in HEK cells, as least for the period tested in this study. However, *EGFP* expression from [*EGFP^circles^*] is unstable and quickly silenced, a pattern similar to *EGFP* expression from transiently transfected plasmids. A previous study has found that transfected plasmids can be asymmetrically segregated in dividing mammalian tissue culture cells (21). Consistent with that, when assaying the median and mean fluorescence intensity in the remaining small proportion of GFP+ cells (Supplementary Figure S20), we found that the GFP+ signal intensity increased at later passages.

### CRISPR-C can generate eccDNAs of various sizes

**Figure 4.**
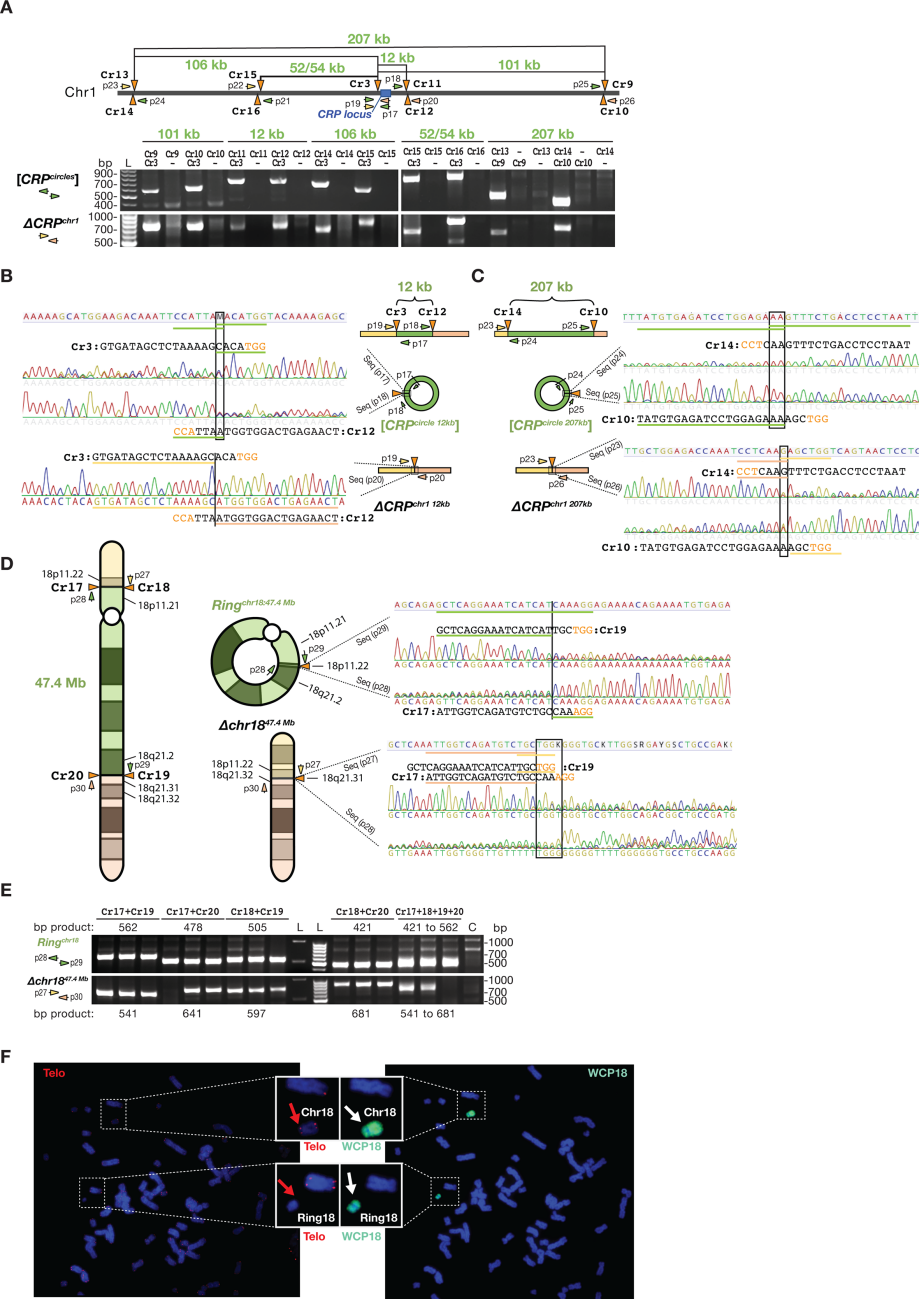
Generation of eccDNAs of various sizes and ring chromosome 18 by CRISPR-C. (**A**, upper part) Schematic view of distances between targeted CRISPR gRNAs on chromosome 1 (Cr9 to Cr17) along with diagnostic oligos (p17 to p26) for PCR confirmation of formation of (A, lower part) kb-sized [*CRP^circles^*] and corresponding *CRP* deletions, Δ*CRP^chr1^*. (**B** and **C**) Chromatograms of Sanger sequencing across the junction of the [*CRP^circle 12 kb^*] and the [*CRP^circle 207 kb^*] in both directions and chromatograms below confirming *CRP* deletions, Δ*CRP^chr1 12 kb^*and Δ*CRP^chr1 207 kb^*. (**D**) Left, schematic view of chromosome 18 with annotated Cr18 to Cr21 sites and resultant formation of *Ring^chr18:47.4 Mb^* and *Δchr18^47.4Mb^* after CRISPR-C and DNA repair. Right, Sanger sequencing chromatograms confirming expected junctions. (**E**) Corresponding gel-images of amplified PCR products across junctions associated to D. Schemes, not drawn to scale. (**F**) Karyotyping ring18-like structure of chromosome 18 by telomere staining (Telo) and whole chromosome 18 painting (WCP18) of HEK293T cells treated with CRISPR-C. Blue, DAPI staining of DNA; Red, telomere; Cyan, chromosome 18.

We next asked whether CRISPR-C also could generate eccDNA in sizes larger than 2 kb. To address this question, we generated several pairs of CRISPR gRNAs targeting chromosome 1 (Figure 4A) to test for eccDNA formation in sizes up to 207 kb. For all examined sites, we confirmed eccDNA formation by CRISPR-C (Figure 4A–C), confirming that CRISPR-C can generate eccDNAs in a range of different sizes. As controls, we further proved that single double-strand DNA breaks by CRISPR gene editing did not led to eccDNA formation (Figure 4A–C) as also observed for similar control experiments with the ECC biosensor (Supplementary Figures S4 and S6) and the [*UPA^circle exon 4^*] (Figure 2G).

### Generation of ring chromosomes by CRISPR-C

Ring chromosomes are chromosomal alterations found in humans and have been reported for all chromosomes, which cause various developmental defects during fetal development and are associated to cancers (22). Ring chromosome 18 is found at high frequency in 1 out of 40 000 births (23). Currently, there is no tool for re-modelling ring chromosomes *in vitro*. We next investigated whether CRISPR-C can be used to generate ring chromosome 18, Ring^chr18^. Two pairs (Cr17+Cr18 and Cr19+Cr20) of CRISPR gRNAs were designed to target the *p* and *q* aims of chromosome 18, respectively. When HEK293T cells were treated with the designed CRISPR-pairs, we detected both efficient formation of large DNA deletions, Δchr18^47.4 Mb^ as well as Ring^chr18:47.4 Mb^. Sanger sequencing of amplified PCR products across the junction validated their structures (Figure 4D and E). Karyotyping, telomere staining and whole chromosome 18 painting of HEK293T cells treated with CRISPR-C further confirmed the appearance of ring18-like structures with an efficiency of ∼2% (2/113 metaphases analyzed, Figure 4F), whereas no ring18-like structures were found in untreated cells (0/65 metaphases analyzed, Supplementary Figure S21). In addition, we observed that cells with the ring18-like genotype had lower fitness, as the ring18-like structure was eventually lost from cell culture when passaged multiple times. This result is not surprising as ring chromosomes are reported to be unstable (24).

## DISCUSSION

We previous demonstrated that CRISPR–Cas9 can be used for efficient and multiplexed genetic manipulations (25–27). Here, we further showed, for the first time, that CRISPR technology can be used efficiently to form specific eccDNA sequences in the human genome. Designated CRISPR-C, eccDNA was formed at all regions tested, if targeted by CRISPR-pairs on the same chromosome. In detail, the introduction of two double-stranded DNA breaks and subsequent DNA repair, mainly driven by blunt end-joining, led to formation of an eccDNA product and the corresponding chromosomal DNA deletion or alternatively to DNA inversion. We demonstrated that CRISPR-C can generate entire ring chromosomes inside cells and eccDNAs can be generated in a broad range of sizes from both genic and intergenic regions. We provided evidence that DNA circularization by CRISPR-C is cell-line independent (Figure 2, Supplementary Figures S13 and S14). Several reports support that eccDNAs can be transcriptionally active (8,11,16,28–30). We consolidated previous observations by showing that cells formed [*EGFP^circles^*], *ΔEGFP* and *EGFP^inv^* genotypes after CRISPR-C (Figure 1), which led to active expression of green and/or red fluorescence proteins.

In this study, we confirmed that the ECC biosensor cassette can be used for studying eccDNA biogenesis in human cells with direct linkage to fluorophore synthesis. The exogenous ECC biosensor cassette can likely be a useful marker for deciphering more about the DNA repair machinery, leading to eccDNA biogenesis. As well, via dual fluorophore transcription, the ECC biosensor can provide more insights to the heterogeneity and the mutational spectrum when using CRISPR-C. The cassette can either be transfected as a plasmid into cells or used for viral transduction to generate stable chromosome integration. In future experiments, e.g. by cell-dilution down to single cells, CRISPR-C induced cells could be analysed by targeted sequencing or propagated mildly, to assess if mainly homogenous or heterogenous genotypes exist.

Only a few studies have previously looked at eccDNA stability over time (30,31). Continuous cultures of CRISPR-C induced stable cells with the ECC biosensor revealed that the GFP+ cell population diluted out proportionally to the cell-split ratio after few cell passages (Figure 3B and C). However, assessed by outward PCR analyses, the presence of [*EGFP^circles^*] diluted out more slowly (Figure 3E and Supplementary Figure S18E), suggesting that [*EGFP^circles^*] could persist in cell populations for much longer time than two weeks. Furthermore, the more moderate decline in [*EGFP^circles^*] levels over time advocated for possible replication and clustering since the GFP+ cell population decreased over time while the mean fluorescence intensity per cells increased significantly (Supplementary Figure S20). Alternatively, continuous *EGFP* transcription in slow growing or quiescence cells could possibly lead to elevated EGFP levels in a subpopulation of cells. Additional studies will be needed to elucidate this matter further. In order to generally assess eccDNA stability in mammalian cells, the ECC biosensor might be a poor representative as many other unknown factors might contribute to the stability, such as size and sequences constitutions (e.g. replication origins, epigenetics, DNA motifs, repeats, genes, etc.).

Replication of acentric eccDNA in dividing cells can quickly led to copy-number variations, if segregated asymmetrically. It remains to be investigated whether the generated eccDNAs in this study might replicate, which could explain longer persistence-time and increased intensity in cells. Previous reports on microscopic visible eccDNAs (double minutes) showed they can replicate at cell division (32–34). Moreover, upon various replicative stresses, increased eccDNA clustering and micronuclei exclusion was observed, which correlated with altered cellular traits (11,29,35).

A link between eccDNA and cellular aging has previously been described in the eukaryotic cell model of budding yeast. As for many other eukaryotes, the ribosomal RNA genes are numerous and highly repetitive in yeast. So-called [rDNA^circles^] (14) or ERCs (36) are frequently produced and ERC accumulation has been associated to cell senescence and aging of yeast (36). A potentially similar accumulation of eccDNAs in human cells could perhaps also moderate the number of possible cell divisions, causing aging. EccDNA that encodes a specific gene has also been shown to improve the growth fitness of yeast (16), confer drug-resistance for more than 75 days in human cell culture (30), and to transmit drug-resistance vertically to progeny of crop weed (37).

In this study, we demonstrated the application of CRISPR-C in generating eccDNA from different loci, of different sizes, and even for generation of a circular chromosome 18. Our study highlights that future studies of chromosomal abnormalities in medical genetics, such as translocation, deletion, gene-fusion and ring chromosomes (38) can now be supplemented by applying CRISPR-C technology. This method is especially useful for cancer research as around half of all tumors carry eccDNAs with oncogenes (10,11,13). We expect that CRISPR-C will improve our understanding of the role of endogenous eccDNAs in cancer development. The ease of generating ring chromosomes by CRISPR-C can further provide easier modelling of human genetic disorders caused by ring chromosomes (22,24). In addition, the fact that ring chromosomes can be generated by CRISPR-C, might provide valuable knowledge and novel concept arts to the design of synthetic chromosomes (39) and synthetic genomes (40).

## DATA AVAILABILITY

The eccDNA biosensor vectors and CRISPR-C plasmids are available at Addgene repository (https://www.addgene.org/Yonglun Luo/). The eccDNA biosensor reporter cells can be requested from Yonglun Luo directly. All detail protocols can be found at www.dream.au.dk or requesting to the corresponding author Yonglun Luo.

## Supplementary Material

Supplementary Data
