# Supplementary material for: CRISPR-C: circularization of genes and chromosome by CRISPR in human cells
Source: Nucleic Acids Res. 2018 Aug 24;46(22):e131. doi: 10.1093/nar/gky767 (PMC6294522; doi:10.1093/nar/gky767)
Supplement: Supplementary Data [file gky767_supplemental_files.pdf]

Supplementary Materials for

Circularization of genes and chromosome by CRISPR in human cells

CRISPR-C: Circularization of genes and chromosome by CRISPR in human cells

Henrik Devitt Møller [1,†], Lin Lin [2,†], Xi Xiang[3,2,5†], Trine Skov Petersen [2], Jinrong Huang [1,4,5,6], Luhan Yang [7], Eigil Kjeldsen [8], Uffe Birk Jensen [2,8], Xiuqing Zhang [3,4,6], Xin Liu [4,5,6], Xun Xu [4,5,6], Jian Wang [4,5,6,9], Huanming Yang [4,5,6,9], George M. Church [7,10], Lars Bolund [2,4,5,11], Birgitte Regenberg [1,*], Yonglun Luo [2,4,5,6,11, †,*,‡]

[1]Department of Biology, Faculty of Science, University of Copenhagen, Denmark

[2]Department of Biomedicine, Aarhus University, Denmark

[3]BGI Education Center, University of Chinese Academy of Sciences, Shenzhen, China

[4]BGI-Shenzhen, Shenzhen 518083, China

[5]BGI-Qingdao, Qingdao 266555, China

[6]China National GeneBank, BGI-Shenzhen, Shenzhen 518120, China

[7]eGenesis, Inc., Cambridge, MA 02139, USA

[8]Department of Clinical Medicine, Aarhus University, Denmark.

[9]James D. Watson Institute of Genome Science, 310008 Hangzhou, China

[10]Department of Genetics, Harvard Medical School, Boston, MA 02115, USA

[11]Lars Bolund Institute of Regenerative Medicine, BGI-Qingdao, Qingdao 266555, China

[†]These authors contributed equally to this work.

[‡]Lead contact.

* To whom correspondence should be addressed: Yonglun Luo, Tel: 0045-87167761; Fax: 0045-87 16 77 61; Email: alun@biomed.au.dk. Correspondence may also be addressed to Birgitte Regenberg Tel: 0045-35 32 16 80; Fax: 0045-35 32 16 80; Email: bregenberg@bio.ku.dk

This PDF file includes:

Supplementary Text
Figs. S1 to S21

Table S1-S2

Supplementary Figure legends

Fig. S1. TRE-ECC biosensor vector map and sequence.

Fig. S2. CAG-ECC biosensor vector map and sequence.

Fig. S3. Model of CAG-ECC biosensor induction by CRISPR-pairs. Mediated by DNA repair, the two simultaneous double-stranded breaks, introduced by transient cell-transfection of CRISPR-pairs Cr1 and Cr2, will lead to formation of [$EGFP^{circle}$] and Δ*EGFP* or to the inversion of *EGFP*.

Fig. S4. Testing the CAG-ECC biosensor vector in HEK293T cells. (A) Graphical map of vector with sites of CRISPR-pairs (Cr1+Cr2). (B) Outline of plasmid assay experiment. (C) Light (LM) and fluorescence microscopy, green (EGFP), red (mCherry). Images (40x) in triplicates of HEK293T cells transfected with CAG-ECC biosensor vector together with Cr1+Cr2. (D) Images of serial sets of control transfection experiments. Tet, tetracycline. Transfection of EGFP plasmid with Cr1+Cr2 served as a positive control for green cells and negative for red cells.

Fig. S5. CAG-ECC biosensor microscopy assay at higher magnification in HEK293T cells. (A) Graphical map of the vector showing CRISPR-pairs (Cr1+Cr2). (B) Outline of plasmid assay experiment. (C) Fluorescence microscopy (100x) in different channels; blue (DAPI), green (EGFP), red (mCherry), merged images. Cr1+Cr2, gRNAs; pUC19, negative control transfection, EGFP plasmid, positive EGFP control; white bar, 50 µm.

Fig. S6. Testing the TRE-ECC biosensor vector in HEK293T cells. (A) Graphical map of vector with sites of CRISPR-pairs (Cr1+Cr2). (B) Outline of plasmid assay experiment. (C) Light (LM) and fluorescence microscopy, green (EGFP), red (mCherry). Images (40x) in triplicates of HEK293T cells transfected with TRE-ECC biosensor vector together with Cr1+Cr2 -/+ tetracycline (Tet). (D) Images of control HEK293T cell transfections.

Fig. S7. TRE-ECC biosensor microscopy assay at higher magnification in HEK293T cells. (A) showing CRISPR-pairs (Cr1+Cr2). (B) Outline of experiment. (C) Fluorescence microscopy (100x) in different channels; blue (DAPI), green (EGFP), red (mCherry), merged images. Cr1+Cr2, gRNAs; pUC19, negative control transfection; Tet, tetracycline; white bar, 50 µm.

Fig. S8. Assessment of cell clones with integrated CAG-ECC biosensor. (A, upper part) Outline of stable genomic integration of pCAG-ECC in HEK293T cells. (B) Copy-number DNA evaluation by Southern blot with EGFP-probe after KpnI digestion. (A, lower part) Outline of experiment for assessment of integrated TRE-ECC by CRISPR gRNAs (Cr1+Cr2). (C) FACS gating images of the nine isolated clones and treatment with Cr1+Cr2. (D) Histograms of fluorescence cell percentages of the nine CAG-ECC clones after CRISPR-C and FACS analysis.

Fig. S9. Integrated TRE-ECC assay of fifteen cell clones. (A, upper part) Outline of stable genomic integration of TRE-ECC in HEK293T cells. (A, lower part) Outline of CRISPR gRNAs (Cr1+Cr2) assay for assessment of integrated TRE-ECC clones. (B-C) FACS gating images of isolated clones after treatment with Cr1+Cr2 in (B) absence or (C) presence of tetracycline (Tet).

Fig. S10. Correlation between percentage of GFP+ or mCherry+ median intensity with copy numbers of ECC biosensor. Correlation was carried for the co-colored cells (GFP+mCherry+), Pearson rank test.

Fig. S11. Validation of CAG-ECC genotypes after dual-CRISPR. (A) PCR and Sanger sequencing test of genotypes formed after dual-CRISPR gRNA (Cr1+Cr2) in CAG-ECC clone 2 and clone 4, confirming

EGFP^{inversion} and [*EGFP*^{circle}] formation. C, negative CRISPR control; NC, non-template control. (B) Southern blot, probed with EGFP on HEK293T purified and digested DNA from untreated (C, *KpnI*) and Cr1+Cr2 treated cells (clone 2). X=*XbaI*, H=*HindIII*.

Fig. S12. Anticipated outcomes after CRISPR-C gene editing. *, junctions sites after dual-CRISPR restriction with specific gRNAs (scissors) and subsequent repair of DNA breakage sites, mediated by non-homologous end-joining or microhomology mediated end-joining.

Fig. S13. EccDNA detection by three different purification methods in HEK293T cells. (A) Map of chromosome 1, 2-3 kb downstream of the *CRP* locus. gRNAs: Cr3, Cr4; diagnostic oligos: p5 to p8. (B) Experimental outline of DNA purification and enrichment of eccDNA by exonuclease (Exo) removal of linear DNA (+, 15U; ++ 90U). (C) Gel-images of corresponding PCR products across the junctions of [*dsCRP*$^{circle\ chr1:159,708\text{-}159,71Mb}$], along with control test for linear DNA (p31+p32) and plasmid DNA (pUC19), using templates before (-Exo) and after exonuclease treatment (+Exo/++Exo) at three different cell concentrations (10^5, 10^4, 10^2 cells). Blue rectangles 1 and 2 represent [*dsCRP*$^{circle\ 1q23.2:0.57\ kb}$] PCR product purified from genomic DNA preparation (G.DNA-prep) and cell lysate treated with exonuclease treatment (++Exo), respectively. (D) Chromatograms from Sanger sequencing of PCR products from the [*dsCRP*$^{circle\ 1q23.2:0.57\ kb}$], blue rectangles 1 and 2. C, negative CRISPR control (HEK293T cell lysate); NC, non-template control.

Fig. S14. Detection of eccDNA by three different purification methods in HMF cells. (A) Map of chromosome 1, 2-3 kb downstream of the *CRP* locus. gRNAs; Cr3, Cr4; diagnostic oligos; p5 to p8. (B) Experimental outline of DNA purification and enrichment of eccDNA by exonuclease (Exo) removal of linear DNA (+, 15U; ++ 90U). (C) Gel-images of corresponding PCR products across the junctions of [*dsCRP*$^{circle\ 1q23.2:0.57\ kb}$], along with control test for linear DNA (p31+p32) and plasmid DNA (pUC19), using templates before (-Exo) and after exonuclease treatment (+Exo/++Exo) at three different cell concentrations (from 10^5 down to 10^2 cells). (D) Chromatograms from Sanger sequencing of PCR products, blue boxes, from purification of [*dsCRP*$^{circle\ 1q23.2:0.57\ kb}$] from genomic DNA preparation (G.DNA-prep) and cell lysate. C, negative CRISPR control (HEK293T cell lysate); NC, non-template control.

Fig. S15. Validation of deletion after CRISPR-pairs at the *UPA* locus. (A) Map of chromosome 10 map at the *PLAU (UPA)* locus. gRNA, Cr5 and Cr6, diagnostic oligos; p9 to p12. (B) Experimental outline. (C) Gel-image of corresponding PCR products across the junctions of the Δ*UPA* and corresponding chromatogram from Sanger sequencing, red box.

Fig. S16. Deletion and inversion validation at the *TRIM28* locus after CRISPR-pairs. (A) Chromosome 19 map at the *TRIM28* locus. gRNA, Cr7 and Cr8, diagnostic oligos; p13 to p16. (B) Experimental outline. (C) PCR confirmation of the Δ*TRIM28* after Cr7+Cr8. (D) PCR and sequencing confirmation of the *TRIM28*^{inversion}. C, negative CRISPR control of HEK293T cell lysate.

Fig. S17. Indel distribution across the junction of [*TRIM28*^{circle}] in HEK293T.

Fig. S18. CAG-ECC time-course of [*EGFP*^{circle}] expression and retention in cell culture. (A) Outline of time-course experiment. (B) Left, percent fluorescence cells from 1 to 6 cell passages (1:3 split-ratio. (C) Corresponding FACS gating images from B. (D) Outward PCR analysis of [*EGFP*^{circles}] at passage 1 to 6. Cr1+Cr2 (1+2), CRISPR gRNAs; C, control – gRNA; ctrl, DNA template control. (E) Quantification of [EGFP^{circles}] by normalizing to *GAPDH* (solid line) using Image J. Theoretical 1/3 dilution curve was presented as dash line.

Fig. S19. Genotyping of Δ*EGFP*, [*EGFP*^{circle}], *EGFP*^{INV} of FACS-sorted ECC reporter cells after CRISPR-C. (A) Presentation of gating examples of FACS-sorted ECC reporter cells after CRISPR-C; P1-

P4 represent the different population of cells. (B) PCR genotyping. *GAPDH* was used as internal control. (C) Quantification of PCR genotyping results from (B) by Image J.

Fig. S20. Dot plots of fluorescent intensity (GFP), GFP+ cell counts in control and CRISPR-C treated ECC reporter cells over 6 passages. Control (CTRL) cells were transfected with a pUC19 control plasmid, and CRISPR-C treated cells were treated with Cr1+Cr2.

Fig. S21. Metaphase spreads and FISH of telomere and whole chromosome 18 painting. Karyotyping of chromosome 18 in wildtype (WT) HEK293T cells and after treatment with CRISPR-pairs (Cr1+Cr2), using telomere staining (Telo) and whole chromosome 18 painting (WCP18). Blue, DAPI; Red, telomere; Cyan, chromosome 18.

Supplementary Tables

Table S1

gRNA name	5'-3' (sense)	3'-5' (anti-sense)	hg38 region	CRISPR target
**Cr1**	CACCGAGAGCCCCAGAGACCGGCAC	AAACGTGCCGGTCTCTGGGGCTCTC	-	ECC-biosensor
**Cr2**	CACCGCTTGAACGCGTCCCGGCTTG	AAACCAAGCCGGGACGCGTTCAAGC	-	ECC-biosensor
**Cr3**	CACCGTGATAGCTCTAAAAGCACA	AAACTGTGCTTTTAGAGCTATCAC	1q23.2	*dsCRP*
**Cr4**	CACCGATAATTGCTTAATCACACA	AAACTGTGTGATTAAGCAATTATC	1q23.2	*dsCRP*
**Cr5**	CACCGTGGCCACACAAATGTGAGG	AAACCCTCACATTTGTGTGGCCAC	10q22.2	*uPA*
**Cr6**	CACCGACAAGTTGGGAAGGCTTCA	AAACTGAAGCCTTCCCAACTTGTC	10q22.2	*uPA*
**Cr7**	CACCGAGAGCGCCTGCGACCCGAG	AAACCTCGGGTCGCAGGCGCTCTC	19q13.43	*TRIM28*
**Cr8**	CACCGTGCTTCTCCAAAGACATCG	AAACCGATGTCTTTGGAGAAGCAC	19q13.43	*TRIM28*
**Cr9**	CACCGAGCTGGTCAGTAACTCCTCT	AAACAGAGGAGTTACTGACCAGCTC	1q23.1	chr1
**Cr10**	CACCGTGAGATCCTGGAGAAAAGC	AAACGCTTTTCTCCAGGATCTCAC	1q23.1	chr1
**Cr11**	CACCGTGGAAGACAAATTCCATTAA	AAACTTAATGGAATTTGTCTTCCAC	1q23.1	chr1
**Cr12**	CACCGAGTTCTCAGTCCACCATTAA	AAACTTAATGGTGGACTGAGAACTC	1q23.1	chr1
**Cr13**	CACCGTAAGGCTTTTTATTGCAGG	AAACCCTGCAATAAAAAGCCTTAC	1q23.1	chr1
**Cr14**	CACCGATTAGGAGGTCAGAAACTTG	AAACCAAGTTTCTGACCTCCTAATC	1q23.1	chr1
**Cr15**	CACCGAAGGTGACCTTCAAATTTGT	AAACACAAATTTGAAGGTCACCTTC	1q23.1	chr1
**Cr16**	CACCGTGGCATACAAGAAATAATGA	AAACTCATTATTTCTTGTATGCCAC	1q23.1	chr1
**Cr17**	CACCGATGGTCAGTGTCTCGTGGGC	AAACGCCCACGAGACACTGACCATc	18p11.22	Ring-chr1 (left arm)
**Cr18**	CACCGATTGGTCAGATGTCTGCCAA	AAACTTGGCAGACATCTGACCAATc	18p11.22	Ring-chr1 (left arm)
**Cr19**	CACCGTGCTGATTACTATGAGGTA	AAACTACCTCATAGTAATCAGCAC	18q21.2	Ring-chr1 (right arm)
**Cr20**	CACCGCTCAGGAAATCATCATTGC	AAACGCAATGATGATTTCCTGAGC	18q21.2	Ring-chr1 (right arm)

Table S2.

PCR target	PCR test	5'- 3' Forward	5'- 3' Reverse	Expected size (bp)	hg38 region	Oligo ID
PLAU (uPA)	Deletion/wt	TACGAACAGGGTGAGGCGAGAG	TGCCAACGGACATTTTAAAGATGTC	258+654	10q22.2	p9 + p12
PLAU (uPA)	EccDNA	AGTGGAAGTGGTAAGGGGTGGC	ATTTGGGGACAGGGAGGGATGG	199	10q22.2	p10+p11
dsCRP_2kb	Deletion/wt	CTCCGTCTTGCACTAGGGGTAT	GGTTGTCGTACTTGCCTGGATT	639 +1232	1q23.2	p8+p5
dsCRP_2kb	EccDNA	TGCTTCTTTAAACGTGACCACCT	GGTCTTCCCCTTAGCCTACTTCT	530	1q23.2	p6+p7
dsCRP_2kb	Inversion	GGTTGTCGTACTTGCCTGGATT	GGTCTTCCCCTTAGCCTACTTCT	453	1q23.2	p5+p7
dsCRP_2kb	Inversion +/-SINE	CTCCGTCTTGCACTAGGGGTAT	TGCTTCTTTAAACGTGACCACCT	756+500	1q23.2	p8+p6
TRIM28	Deletion/wt	AGCCTCGGCCTCTGCCTCAGCC	GTTCGCATCCTGGGCGTCGGTG	171+661	19q13.43	p13+p16
TRIM28	EccDNA	TTGTAACAGTCTCCCACATCCCT	TCGTATGCAGGACCCAGAAAGTA	297	19q13.43	p14+p15
TRIM28	Inversion	TCGTATGCAGGACCCAGAAAGTA	GTTCGCATCCTGGGCGTCGGTG	340	19q13.43	p15+ p35
pUC19	Plasmid control	CTGCAGGTCGACTCTAGAGGAT	CCATTCAGGCTGCGCAACTG	195	-	p33+ p34
EGFP (ECC biosensor)	Deletion/wt	GTTATCCTCCTCGCCCTTGC	TCCGTACCACTTCCTACCCT	142+2059	-	p4+p0
EGFP (ECC biosensor)	EccDNA	AAGGGATGGTTGGTTGGTGG	GTGCTGCTTCATGTGGTCGG	361	-	p1+p3
EGFP (ECC biosensor)	Inversion	AACAGCTCCTCGCCCTTG	TCCGTACCACTTCCTACCCT	127	-	p2+p0
mCherry (ECC biosensor)	Inversion	AAGGGATGGTTGGTTGGTGG	GTTATCCTCCTCGCCCTTGC	156	-	p1+p4
dsCRP_106kb	EccDNA	TGCTTTATGTCACATTGCTTTGC	TGCTTCTTTAAACGTGACCACCT	666/756	1q23.1	P24+P19
dsCRP_106kb	Linear DNA	CACAAAAGGGCGTAGAGTTACC	GGTTGTCGTACTTGCCTGGATT	591/500	1q23.1	P23+P17
dsCRP_52kb	EccDNA	TCAAGGTCTTTTATACCCAAGTCA	TGCTTCTTTAAACGTGACCACCT	617	1q23.1	P21+P17
dsCRP_52kb	Linear DNA	AGACGCCTTCTGTCACATCC	GGTTGTCGTACTTGCCTGGATT	788	1q23.1	P22+P17
dsCRP_54kb	EccDNA	CATCTGGGTCTAGCGCATTT	TGCTTCTTTAAACGTGACCACCT	785	1q23.1	P21+P17
dsCRP_54kb	Linear DNA	GACATTCTTCCTCTACCTCCCC	GGTTGTCGTACTTGCCTGGATT	833	1q23.1	P22+P17
upCRP_101kb	EccDNA	GGTTGTCGTACTTGCCTGGATT	TTAGTCCATCTGTCCACTGAGC	724/707	1q23.1	P17+P25
upCRP_101kb	Linear DNA	TGCTTCTTTAAACGTGACCACCT	ATTAACTGTCCTTCCATGTGCC	615/635	1q23.1	P19+P26
upCRP_12kb	EccDNA	GGTTGTCGTACTTGCCTGGATT	TCAAACACTATGGGAATGAGGC	672/680	1q23.1	P19+P20
upCRP_12kb	Linear DNA	TGCTTCTTTAAACGTGACCACCT	GTGACCCCTTAATCAATCTCTCC	763/763	1q23.1	P17+P18
CRP_207kb	EccDNA	TGCTTTATGTCACATTGCTTTGC	TTAGTCCATCTGTCCACTGAGC	589/686	1q23.1	P24+P25
CRP_207kb	Linear DNA	CACAAAAGGGCGTAGAGTTACC	ATTAACTGTCCTTCCATGTGCC	573/479	1q23.1	P23+P26
Ring-chrom18	EccDNA	CATGGAAGTTTGGACAACAATGCTG	AGGATTACTCTGGTGACCTGGCATG	562/478/505/421	18p11.22-18q21.2	P28+P29
Ring-chrom18	Linear DNA	CTCCCAACAAACAGCCTGAGCGTCC	ATCAGCACAGCACCATCTTAGGTAC	541/641/597/681	18p11.22-18q21.2	P27+P30

wt=wildtype; up=upstream; ds=downstream

Fig. S1

pCAG-ECC biosensor vector

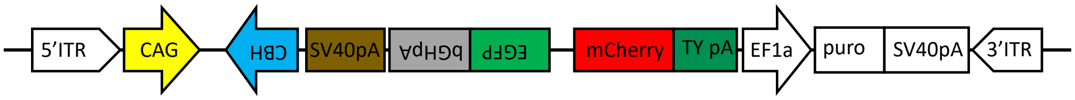

5'…GACATTGATTATTGACTAGTTATTAATAGTAATCAATTACGGGGTCATTAGTTCATAGCCCATATATGGAGTTCCGCGTTACATAACTTACGGT
AAATGGCCCGCCTGGCTGACCGCCCAACGACCCCCGCCCATTGACGTCAATAATGACGTATGTTCCCATAGTAACGCCAATAGGGACTTTCCATTGA
CGTCAATGGGTGGAGTATTTACGGTAAACTGCCCACTTGGCAGTACATCAAGTGTATCATATGCCAAGTACGCCCCCTATTGACGTCAATGACGGTA
AATGGCCCGCCTGGCATTATGCCCAGTACATGACCTTATGGGACTTTCCTACTTGGCAGTACATCTACGTATTAGTCATCGCTATTACCATGGTCGA
GGTGAGCCCCACGTTCTGCTTCACTCTCCCCATCTCCCCCCCCTCCCCACCCCCAATTTTGTATTTATTTATTTTTTAATTATTTTGTGCAGCGATG
GGGGCGGGGGGGGGGGGGGGCGCGCGCCAGGCGGGGCGGGGCGGGGCGAGGGGCGGGGCGGGGCGAGGCGGAGAGGTGCGGCGGCAGCCAATCAGA
GCGGCGCGCTCCGAAAGTTTCCTTTTATGGCGAGGCGGCGGCGGCGGCGGCCCTATAAAAAGCGAAGCGCGCGGCGGGCGGGAGTCGCTGCGACGC
TGCCTTCGCCCCGTGCCCCGCTCCGCCGCCGCCTCGCGCCGCCCGCCCCGGCTCTGACTGACCGCGTTACTCCCACAGGTGAGCGGGCGGGACGGCC
CTTCTCCTCCGGGCTGTAATTAGCGCTTGGTTTAATGACGGCTTGTTTCTTTTCTGTGGCTGCGTGAAAGCCTTGAGGGGCTCCGGGAGGGCCCTTT
GTGCGGGGGGAGCGGCTCGGGGGGTGCGTGCGTGTGTGTGTGCGTGGGGAGCGCCGCGTGCGGCTCCGCGCTGCCCGGCGGCTGTGAGCGCTGCGGG
CGCGGCGCGGGGCTTTGTGCGCTCCGCAGTGTGCGCGAGGGGAGCGCGGCCGGGGGCGGTGCCCCGCGGTGCGGGGGGGGCTGCGAGGGGAACAAAG
GCTGCGTGCGGGGTGTGTGCGTGGGGGGGTGAGCAGGGGGTGTGGGCGCGTCGGTCGGGCTGCAACCCCCCCTGCACCCCCCTCCCCGAGTTGCTGA
GCACGGCCCGGCTTCGGGTGCGGGGCTCCGTACGGGGCGTGGCGCGGGGCTCGCCGTGCCGGGCGGGGGGTGGCGGCAGGTGGGGGTGCCGGGCGGG
GCGGGGCCGCCTCGGGCCGGGGAGGGCTCGGGGGAGGGGCGCGGCGGCCCCCGGAGCGCCGGCGGCTGTCGAGGCGCGGCGAGCCGCAGCCATTGCC
TTTTATGGTAATCGTGCGAGAGGGCGCAGGGACTTCCTTTGTCCCAAATCTGTGCGGAGCCGAAATCTGGGAGGCGCCGCCGCACCCCCTCTAGCGG
GCGCGGGGCGAAGCGGTGCGGCGCCGGCAGGAAGGAAATGGGCGGGGAGGGCCTTCGTGCGTCGCCGCGCCGCCGTCCCCTTCTCCCTCTCCAGCCT
CGGGGCTGTCCGCGGGGGGACGGCTGCCTTCGGGGGGGACGGGGCAGGGCGGGGTTCGGCTTCTGGCGTGTGACCGGCGGCTCTAGAGCCTCTGCTA
ACCATGTTCATGCCTTCTTCTTTTTCCTACAGCTCCTGGGCAACGTGCTGGTTATTGTGCTGTCTCATCATTTTGGCAAAGAATCTAGATACCCTCG
TAAAGGTCTAGAGCTAGCGAATTCGGGGCACAGAGCCCCAGAGACCGGCACCGGTCCAACCTGAAAAAAGTGATTTCAGGCAGGTGCTCCAGGTAA
TTAAACATTAATACCCCACCAACCAACCATCCCTTAAACCCTTACCTCTTGCTCAGCTAATTACAGCCCGGAGGAGAAGGGCCGTCCCGCCCGCTCA
CCTGTGGGAGTAACGCGGTCAGTCAGAGCCGGGGCGGGCGGCGCGAGGCGGCGGCGGAGCGGGGCACGGGGCGAAGGCAGCGTCGCAGCGACTCCCG
CCCGCCGCGCGCTTCGCTTTTTATAGGGCCGCCGCCGCCGCCGCCTCGCCATAAAAGGAAACTTTCGGAGCGCGCCGCTCTGATTGGCTGCCGCCGC
ACCTCTCCGCCTCGCCCCGCCCCGCCCCTCGCCCCGCCCCGCCCCGCCTGGCGCGCGCCCCCCCCCCCCCCCCGCCCCCATCGCTGCACAAAATAAT
TAAAAAATAAATAAATACAAAATTGGGGGTGGGGAGGGGGGGGAGATGGGGAGAGTGAAGCAGAACGTGGGGCTCACCTCGACCATGGTAATAGCGA
TGACTAATACGTAGATGTACTGCCAAGTAGGAAAGTCCCATAAGGTCATGTACTGGGCACAATGCCAGGCGGGCCATTTACCGTCATTGACGTCAAT
AGGGGGCGTACTTGGCATATGATACACTTGATGTACTGCCAAGTGGGCAGTTTACCGTAAATACTCCACCCATTGACGTCAATGGAAAGTCCCTATT
GGCGTTACTATTGACGTCAATGGGCGGGGGTCGTTGGGCGGTCAGCCAGGCGGGCCATTTACCGTAAGTTATGTAACGGGTACCTCTAGAGCCATTT
GTCTGCAATAAAGCAATAGCATCACAAATTTCACAAATAAAGCATTTTTTTCACTGCCCATAGAGCCCACCGCATCCCCAGCATGCCTGCTATTGTC
TTCCCAATCCTCCCCCTTGCTGTCCTGCCCCACCCCACCCCCCAGAATAGAATGACACCTACTCAGACAATGCGATGCAATTTCCTCATTTTATTAG
GAAAGGACAGTGGGAGTGGCACCTTCCAGGGTCAAGGAAGGCACGGGGGAGGGGCAAACAACAGATGGCTGGCAACTAGAAGGCACAGTCGAGGCTG
ATCGGCCGCTCTAGAACTAGTGGATCCCCCGGGCTGCATCCAACCTCGAGTTACTTGTACAGCTCGTCCATGCCGAGAGTGATCCCGGCGGCGGTCA
CGAACTCCAGCAGGACCATGTGATCGCGCTTCTCGTTGGGGTCTTTGCTCAGGGCGGACTGGGTGCTCAGGTAGTGGTTGTCGGGCAGCAGCACGGG
GCCGTCGCCGATGGGGGTGTTCTGCTGGTAGTGGTCGGCGAGCTGCACGCTGCCGTCCTCGATGTTGTGGCGGATCTTGAAGTTCACCTTGATGCCG
TTCTTCTGCTTGTCGGCCATGATATAGACGTTGTGGCTGTTGTAGTTGTACTCCAGCTTGTGCCCCAGGATGTTGCCGTCCTCCTTGAAGTCGATGC
CCTTCAGCTCGATGCGGTTCACCAGGGTGTCGCCCTCGAACTTCACCTCGGCGCGGGTCTTGTAGTTGCCGTCGTCCTTGAAGAAGATGGTGCGCTC
CTGGACGTAGCCTTCGGGCATGGCGGACTTGAAGAAGTCGTGCTGCTTCATGTGGTCGGGGTAGCGGCTGAAGCACTGCACGCCGTAGGTCAGGGTG
GTCACGAGGGTGGGCCAGGGCACGGGCAGCTTGCCGGTGGTGCAGATGAACTTCAGGGTCAGCTTGCCGTAGGTGGCATCGCCCTCGCCCTCGCCGG
ACACGCTGAACTTGTGGCCGTTTACGTCGCCGTCCAGCTCGACCAGGATGGGCACCACCCCGGTGAACAGCTCCTCGCCCTTGCTCACCATGGTGGC
GGTGCCCGGGAGACGCCAACCGCAAGCCGGGACGCGTTCAAGCTTCGCCTGGAGACGATCCGCCGCCACCatggtgagcaagggcgaggaggataac
atggccatcatcaaggagttcatgcgcttcaaggtgcacatggagggctccgtgaacggccacgagttcgagatcgagggcgagggcgagggccgcc
cctacgagggcacccagaccgccaagctgaaggtgaccaagggtggccccctgcccttcgcctgggacatcctgtcccctcagttcatgtacggctc
caaggcctacgtgaagcaccccgccgacatccccgactacttgaagctgtccttccccgagggcttcaagtgggagcgcgtgatgaacttcgaggac
ggcggcgtggtgaccgtgacccaggactcctccctgcaggacggcgagttcatctacaaggtgaagctgcgcggcaccaacttcccctccgacggcc
ccgtaatgcagaagaagaccatgggctgggaggcctcctccgagcggatgtaccccgaggacggcgccctgaagggcgagatcaagcagaggctgaa
gctgaaggacggcggccactacgacgctgaggtcaagaccacctacaaggccaagaagcccgtgcagctgcccggcgcctacaacgtcaacatcaag
ttggacatcacctcccacaacgaggactacaccatcgtggaacagtacgaacgcgccgagggccgccactccaccggcggcatggacgagctgtaca
agtaaGTTTAAACGGGGGAGGCTAACTGAAACACGGAAGGAGACAATACCGGAAGGAACCCGCGCTATGACGGCAATAAAAAGACAGAATAAAACGC
ACGGGTGTTGGGTCGTTTGTTCATAAACGCGGGGTTCGGTCCCAGGGCTGGCACTCTGTCGATACCCCACCGAGACCCCATTGGGGCCAATACGCCC
GCGTTTCTTCCTTTTCCCCACCCCACCCCCCAAGTTCGGGTGAAGGCCCAGGGCTCGCAGCCAACGTCGGGGCGGCAGGCCCTGCCATAGC…3'

Cr1

Cr2

Fig. S2

pTRE-ECC biosensor vector

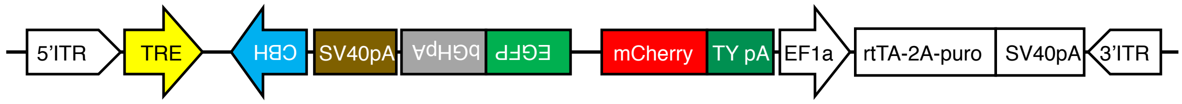

5'…TTTACTCCCTATCAGTGATAGAGAACGTATGAAGAGTTTACTCCCTATCAGTGATAGAGAACGTATGCAGACTTTACTCCCTATCAGTGATAGA
GAACGTATAAGGAGTTTACTCCCTATCAGTGATAGAGAACGTATGACCAGTTTACTCCCTATCAGTGATAGAGAACGTATCTACAGTTTACTCCCTA
TCAGTGATAGAGAACGTATATCCAGTTTACTCCCTATCAGTGATAGAGAACGTATAAGCTTTAGGCGTGTACGGTGGGCGCCTATAAAAGCAGAGCT
CGTTTAGTGAACCGTCAGATCGCCTGGAGCAATTCCACAACACTTTTGTCTTATACCAACTTTCCGTACCACTTCCTACCCTCGTAAAGGTCTAGAG
CTAGCGAATTCGGGGCACAGAGCCCCAGAGACCGGCACCGGTCCAACCTGAAAAAAAGTGATTTCAGGCAGGTGCTCCAGGTAATTAAACATTAATA
CCCCACCAACCAACCATCCCTTAAACCCTTACCTCTTGCTCAGCTAATTACAGCCCGGAGGAGAAGGGCCGTCCCGCCCGCTCACCTGTGGGAGTAA
CGCGGTCAGTCAGAGCCGGGGCGGGCGGCGCGAGGCGGCGGCGGAGCGGGGCACGGGGCGAAGGCAGCGTCGCAGCGACTCCCGCCCGCCGCGCGCT
TCGCTTTTTATAGGGCCGCCGCCGCCGCCGCCTCGCCATAAAAGGAAACTTTCGGAGCGCGCCGCTCTGATTGGCTGCCGCCGCACCTCTCCGCCTC
GCCCCGCCCCGCCCCTCGCCCCGCCCCGCCCCGCCTGGCGCGCGCCCCCCCCCCCCCCGCCCCCATCGCTGCACAAAATAATTAAAAAATAAATA
AATACAAAATTGGGGGTGGGGAGGGGGGGGAGATGGGGAGAGTGAAGCAGAACGTGGGGCTCACCTCGACCATGGTAATAGCGATGACTAATACGTA
GATGTACTGCCAAGTAGGAAAGTCCCATAAGGTCATGTACTGGGCACAATGCCAGGCGGGCCATTTACCGTCATTGACGTCAATAGGGGGCGTACTT
GGCATATGATACACTTGATGTACTGCCAAGTGGGCAGTTTACCGTAAATACTCCACCCATTGACGTCAATGGAAAGTCCCTATTGGCGTTACTATTG
ACGTCAATGGGCGGGGGTCGTTGGGCGGTCAGCCAGGCGGGCCATTTACCGTAAGTTATGTAACGGGTACCTCTAGAGCCATTTGTCTGCAATAAAG
CAATAGCATCACAAATTTCACAAATAAAGCATTTTTTTCACTGCCCATAGAGCCCACCGCATCCCCAGCATGCCTGCTATTGTCTTCCCAATCCTCC
CCCTTGCTGTCCTGCCCCACCCCACCCCCCAGAATAGAATGACACCTACTCAGACAATGCGATGCAATTTCCTCATTTTATTAGGAAAGGACAGTGG
GAGTGGCACCTTCCAGGGTCAAGGAAGGCACGGGGGAGGGGCAAACAACAGATGGCTGGCAACTAGAAGGCACAGTCGAGGCTGATCGGCCGCTCTA
GAACTAGTGGATCCCCCGGGCTGCATCCAACCTCGAGTTACTTGTACAGCTCGTCCATGCCGAGAGTGATCCCGGCGGCGGTCACGAACTCCAGCAG
GACCATGTGATCGCGCTTCTCGTTGGGGTCTTTGCTCAGGGCGGACTGGGTGCTCAGGTAGTGGTTGTCGGGCAGCAGCACGGGGCCGTCGCCGATG
GGGGTGTTCTGCTGGTAGTGGTCGGCGAGCTGCACGCTGCCGTCCTCGATGTTGTGGCGGATCTTGAAGTTCACCTTGATGCCGTTCTTCTGCTTGT
CGGCCATGATATAGACGTTGTGGCTGTTGTAGTTGTACTCCAGCTTGTGCCCCAGGATGTTGCCGTCCTCCTTGAAGTCGATGCCCTTCAGCTCGAT
GCGGTTCACCAGGGTGTCGCCCTCGAACTTCACCTCGGCGCGGGTCTTGTAGTTGCCGTCGTCCTTGAAGAAGATGGTGCGCTCCTGGACGTAGCCT
TCGGGCATGGCGGACTTGAAGAAGTCGTGCTGCTTCATGTGGTCGGGGTAGCGGCTGAAGCACTGCACGCCGTAGGTCAGGGTGGTCACGAGGGTGG
GCCAGGGCACGGGCAGCTTGCCGGTGGTGCAGATGAACTTCAGGGTCAGCTTGCCGTAGGTGGCATCGCCCTCGCCCTCGCCGGACACGCTGAACTT
GTGGCCGTTTACGTCGCCGTCCAGCTCGACCAGGATGGGCACCACCCCGGTGAACAGCTCCTCGCCCTTGCTCACCATGGTGGCGGTGCCCGGAGA
CGCCAACCGCAAGCCGGGACGCGTTCAAGCTTCGCCTGGAGACGATCCGCCGCCACCatggtgagcaagggcgaggaggataacatggccatcatca
aggagttcatgcgcttcaaggtgcacatggagggctccgtgaacggccacgagttcgagatcgagggcgagggcgagggccgcccctacgagggcac
ccagaccgccaagctgaaggtgaccaagggtggccccctgcccttcgcctgggacatcctgtcccctcagttcatgtacggctccaaggcctacgtg
aagcaccccgccgacatccccgactacttgaagctgtccttccccgagggcttcaagtgggagcgcgtgatgaacttcgaggacggcggcgtggtga
ccgtgacccaggactcctccctgcaggacggcgagttcatctacaaggtgaagctgcgcggcaccaacttcccctccgacggccccgtaatgcagaa
gaagaccatgggctgggaggcctcctccgagcggatgtaccccgaggacggcgccctgaagggcgagatcaagcagaggctgaagctgaaggacggc
ggccactacgacgctgaggtcaagaccacctacaaggccaagaagcccgtgcagctgcccggcgcctacaacgtcaacatcaagttggacatcacct
cccacaacgaggactacaccatcgtggaacagtacgaacgcgccgagggccgccactccaccggcggcatggacgagctgtacaagtaaGTTTAAAC
GGGGGAGGCTAACTGAAACACGGAAGGAGACAATACCGGAAGGAACCCGCGCTATGACGGCAATAAAAAGACAGAATAAAACGCACGGTGTTGGGT
CGTTTGTTCATAAACGCGGGGTTCGGTCCCAGGGCTGGCACTCTGTCGATACCCCACCGAGACCCCATTGGGGCCAATACGCCCGCGTTTCTTCCTT
TTCCCCACCCCACCCCCCAAGTTCGGGTGAAGGCCCAGGGCTCGCAGCCAACGTCGGGGCGGCAGGCCCTGCCATAGC…3'

Cr1

Cr2

Fig. S3

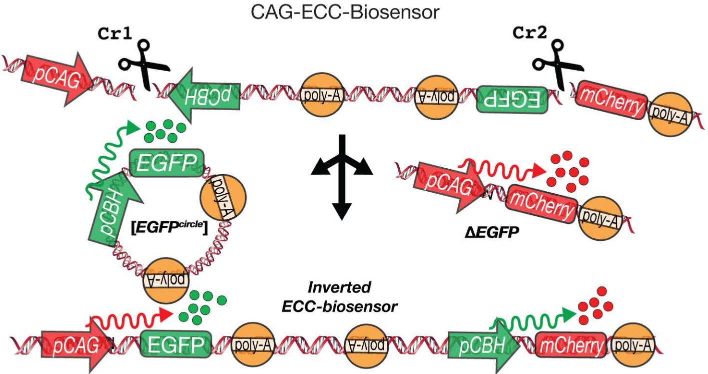

CAG-ECC-Biosensor
Cr1
Cr2
pCAG
pCBH
poly-A
poly-A
EGFP
mCherry
poly-A
EGFP
poly-A
pCBH
[EGFPcircle]
poly-A
pCAG
mCherry
poly-A
ΔEGFP
Inverted
ECC-biosensor
pCAG
EGFP
poly-A
poly-A
pCBH
mCherry
poly-A

Fig. S4

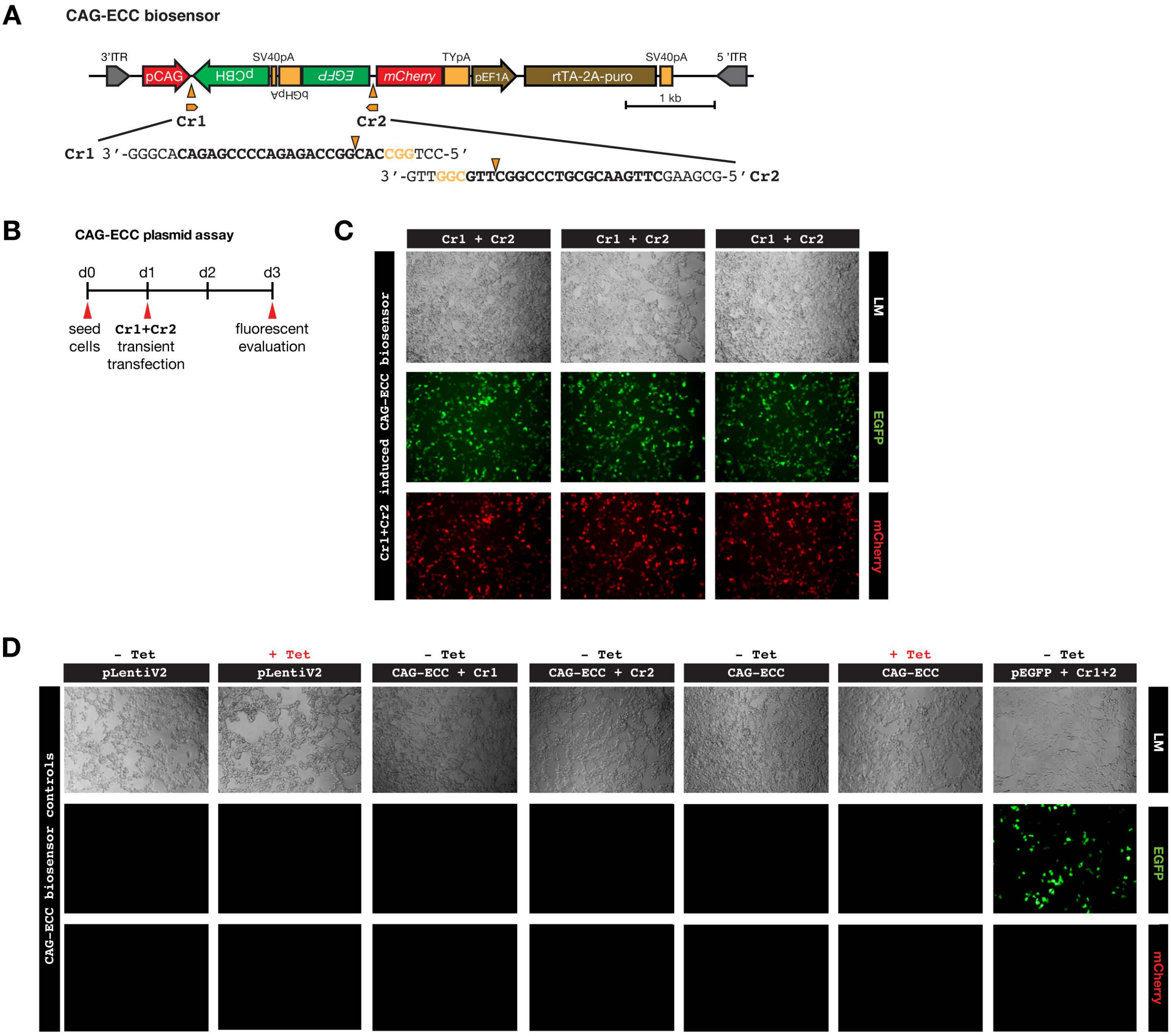
A
CAG-ECC biosensor
3'ITR
pCAG
pCBH
SV40pA
bGHpA
EGFP
mCherry
TYpA
pEF1A
rtTA-2A-puro
SV40pA
5 'ITR
1 kb
Cr1
Cr2
Cr1 3'-GGGCACAGAGCCCCAGAGACCGGCACCGGTCC-5'
3'-GTTGGCGTTCGGCCCTGCGCAAGTTCGAAGCG-5' Cr2
B
CAG-ECC plasmid assay
d0
d1
d2
d3
seed cells
Cr1+Cr2 transient transfection
fluorescent evaluation
C
Cr1 + Cr2
Cr1 + Cr2
Cr1 + Cr2
Cr1+Cr2 induced CAG-ECC biosensor
LM
EGFP
mCherry
D
- Tet
pLentiV2
+ Tet
pLentiV2
- Tet
CAG-ECC + Cr1
- Tet
CAG-ECC + Cr2
- Tet
CAG-ECC
+ Tet
CAG-ECC
- Tet
pEGFP + Cr1+2
CAG-ECC biosensor controls
LM
EGFP
mCherry

Fig. S5

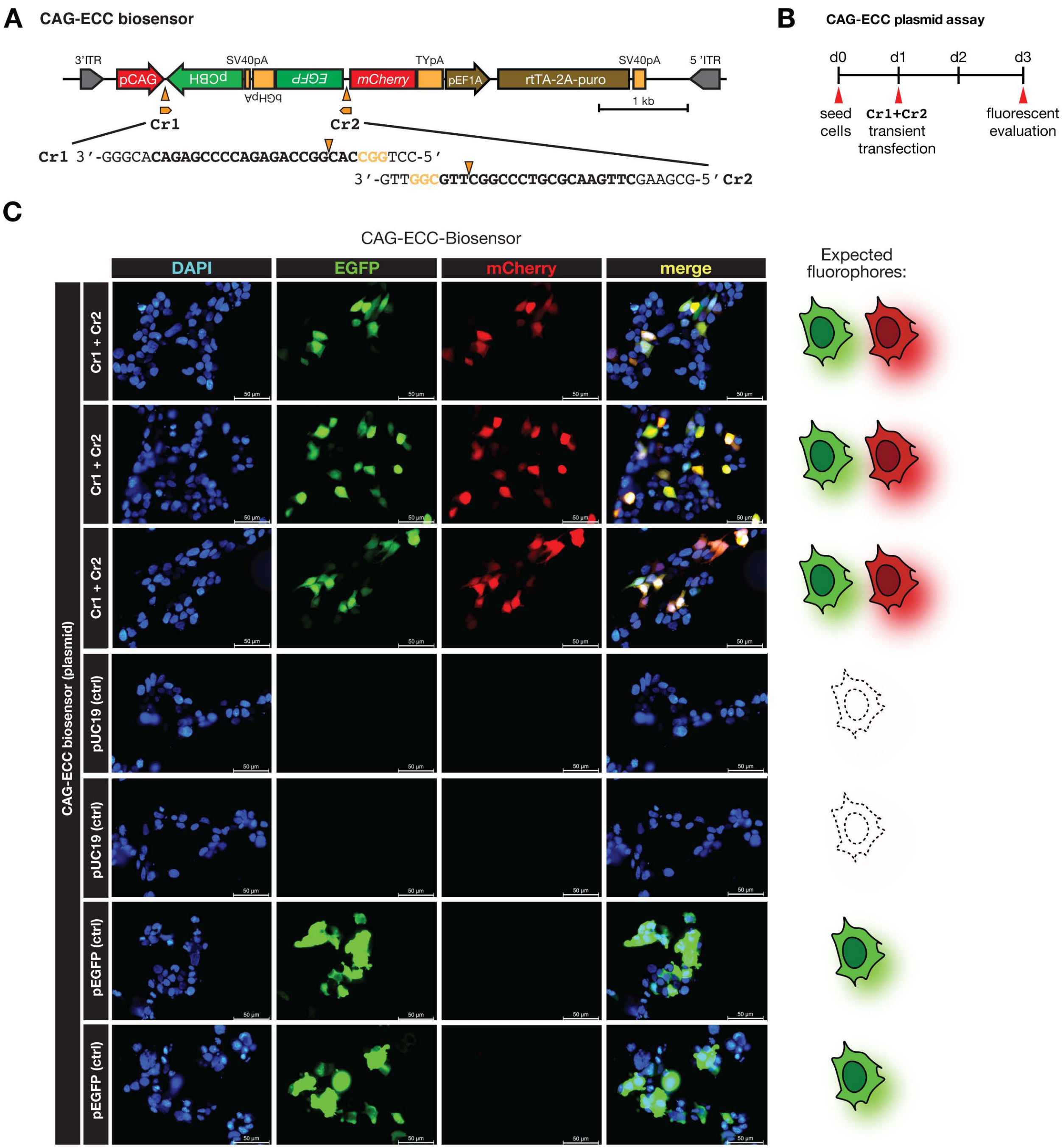
A CAG-ECC biosensor
3'ITR
pCAG
pCBH
SV40pA
EGFP
bGHpA
mCherry
TYpA
pEF1A
rtTA-2A-puro
SV40pA
5 'ITR
1 kb
Cr1
Cr2
Cr1 3'-GGGCACAGAGCCCCAGAGACCGGCACCGGTCC-5'
3'-GTTGGCGTTCGGCCCTGCGCAAGTTCGAAGCG-5' Cr2
B CAG-ECC plasmid assay
d0
d1
d2
d3
seed cells
Cr1+Cr2 transient transfection
fluorescent evaluation
C
CAG-ECC-Biosensor
DAPI
EGFP
mCherry
merge
Expected fluorophores:
CAG-ECC biosensor (plasmid)
Cr1 + Cr2
Cr1 + Cr2
Cr1 + Cr2
pUC19 (ctrl)
pUC19 (ctrl)
pEGFP (ctrl)
pEGFP (ctrl)
50 µm

Fig. S6

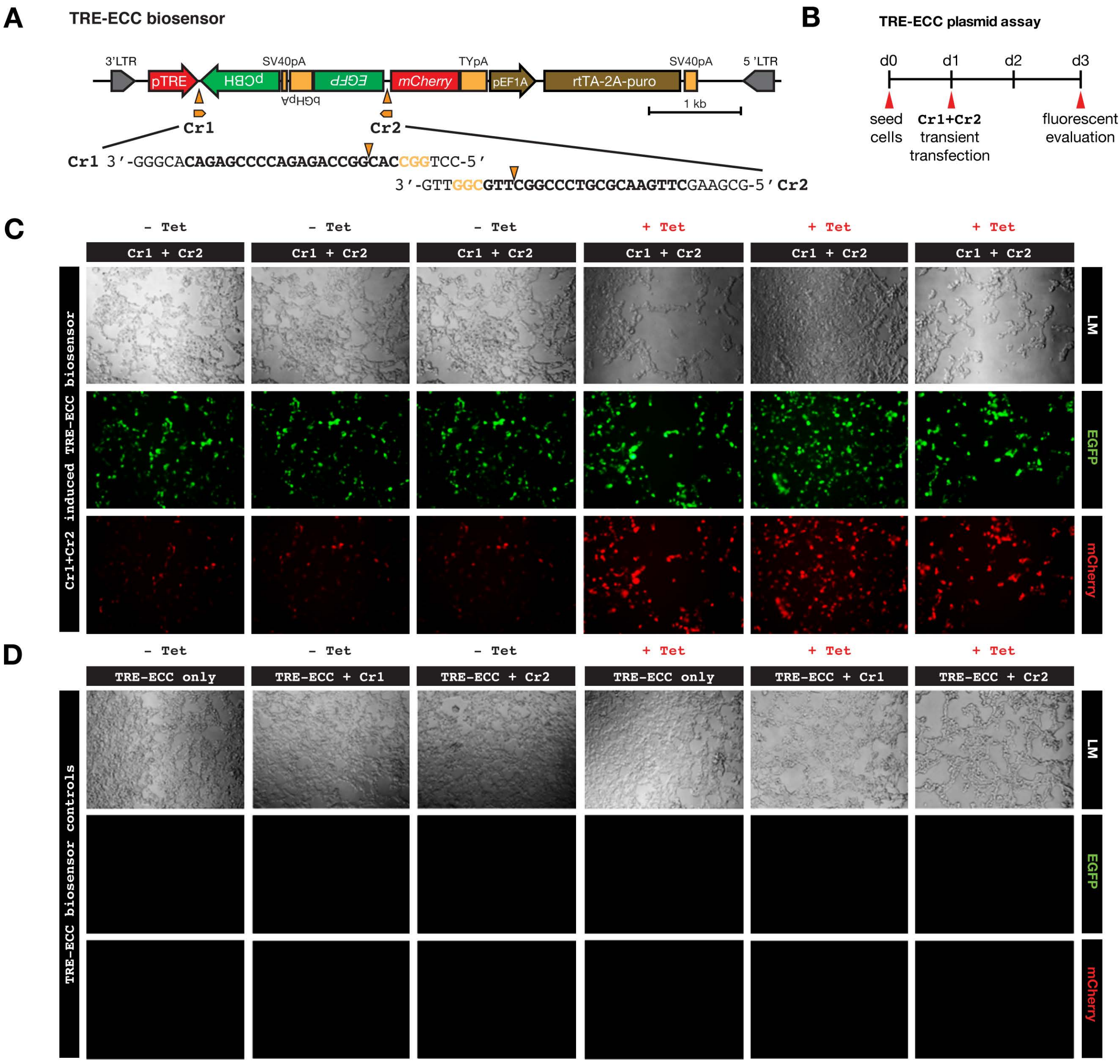
A
TRE-ECC biosensor
3'LTR
pTRE
pCBH
SV40pA
bGHpA
EGFP
mCherry
TYpA
pEF1A
rtTA-2A-puro
SV40pA
5 'LTR
1 kb
Cr1
Cr2
Cr1 3'-GGGCACAGAGCCCCAGAGACCGGCACCGGTCC-5'
3'-GTTGGCGTTCGGCCCTGCGCAAGTTCGAAGCG-5' Cr2
B
TRE-ECC plasmid assay
d0
d1
d2
d3
seed cells
Cr1+Cr2 transient transfection
fluorescent evaluation
C
- Tet
+ Tet
Cr1 + Cr2
Cr1+Cr2 induced TRE-ECC biosensor
LM
EGFP
mCherry
D
- Tet
+ Tet
TRE-ECC only
TRE-ECC + Cr1
TRE-ECC + Cr2
TRE-ECC biosensor controls
LM
EGFP
mCherry

Fig. S7

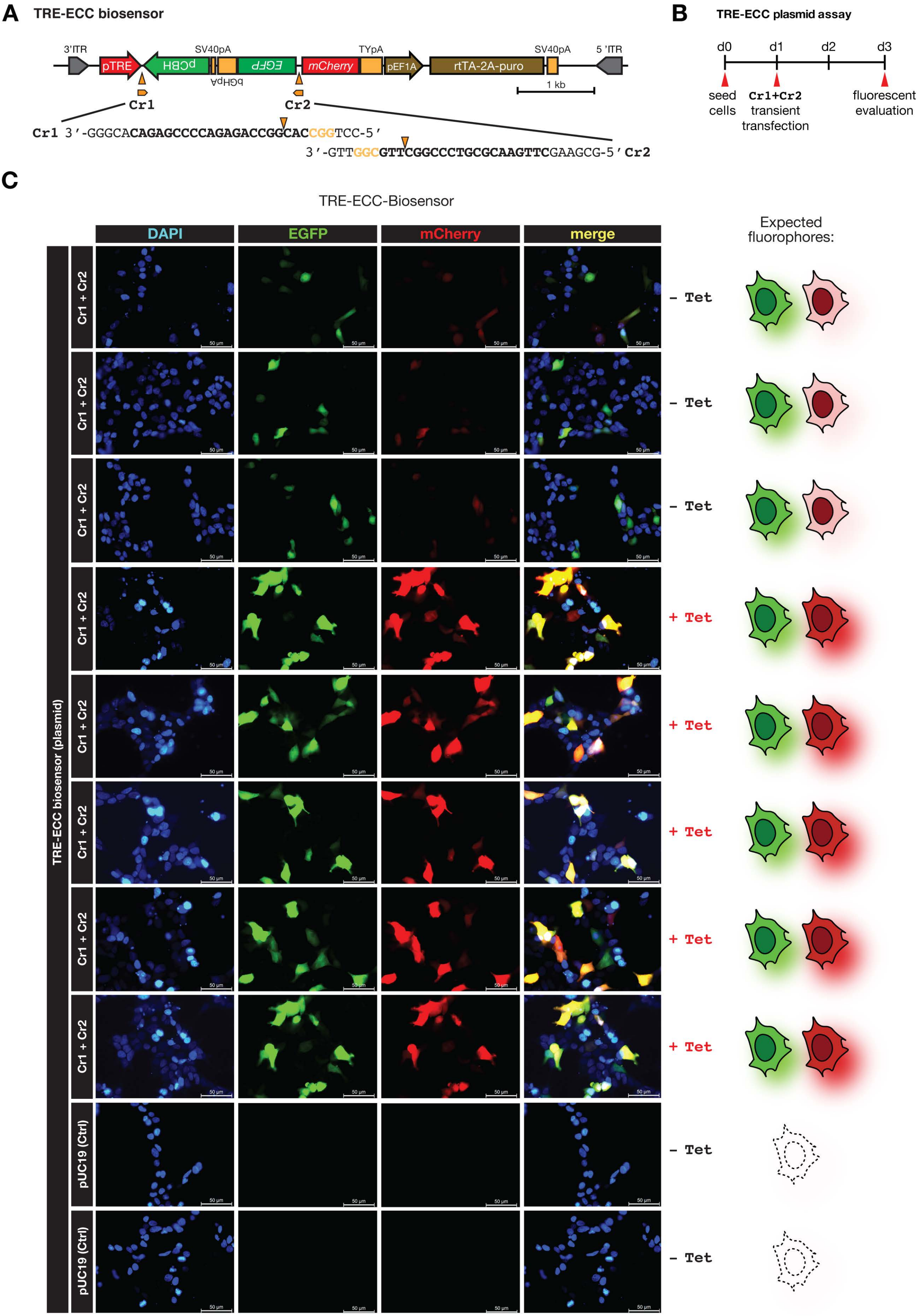
A
TRE-ECC biosensor
3'ITR
pTRE
pCBH
bGHpA
EGFP
SV40pA
mCherry
TYpA
pEF1A
rtTA-2A-puro
SV40pA
5 'ITR
1 kb
Cr1
Cr2
Cr1 3'-GGGCACAGAGCCCCAGAGACCGGCACCGGTCC-5'
3'-GTTGGCGTTCGGCCCTGCGCAAGTTCGAAGCG-5' Cr2
B
TRE-ECC plasmid assay
d0
d1
d2
d3
seed cells
Cr1+Cr2 transient transfection
fluorescent evaluation
C
TRE-ECC-Biosensor
DAPI
EGFP
mCherry
merge
Expected fluorophores:
TRE-ECC biosensor (plasmid)
Cr1 + Cr2
- Tet
+ Tet
pUC19 (Ctrl)
50 µm

Fig. S8

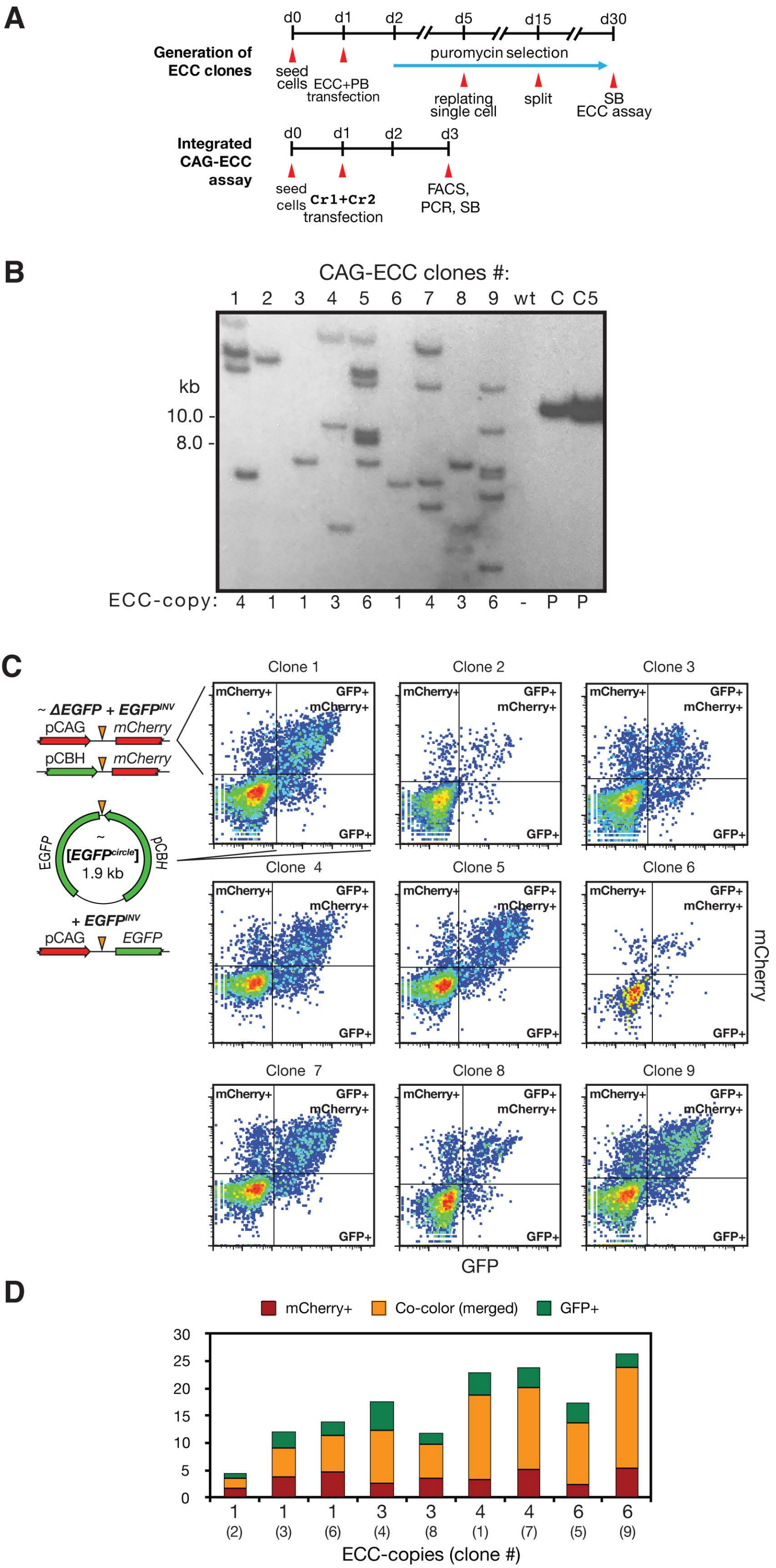
A
Generation of ECC clones
d0
d1
d2
d5
d15
d30
puromycin selection
seed cells
ECC+PB transfection
replating single cell
split
SB ECC assay
Integrated CAG-ECC assay
d0
d1
d2
d3
seed cells
Cr1+Cr2 transfection
FACS, PCR, SB
B
CAG-ECC clones #:
1 2 3 4 5 6 7 8 9 wt C C5
kb
10.0 -
8.0 -
ECC-copy: 4 1 1 3 6 1 4 3 6 - P P
C
~ ΔEGFP + EGFP^INV
pCAG
mCherry
pCBH
mCherry
EGFP
[EGFP^circle]
1.9 kb
pCBH
+ EGFP^INV
pCAG
EGFP
Clone 1
Clone 2
Clone 3
Clone 4
Clone 5
Clone 6
Clone 7
Clone 8
Clone 9
mCherry+
GFP+ mCherry+
GFP+
mCherry
GFP
D
mCherry+
Co-color (merged)
GFP+
30
25
20
15
10
5
0
1 (2)
1 (3)
1 (6)
3 (4)
3 (8
4 (1)
4 (7)
6 (5)
6 (9)
ECC-copies (clone #)

Fig. S9

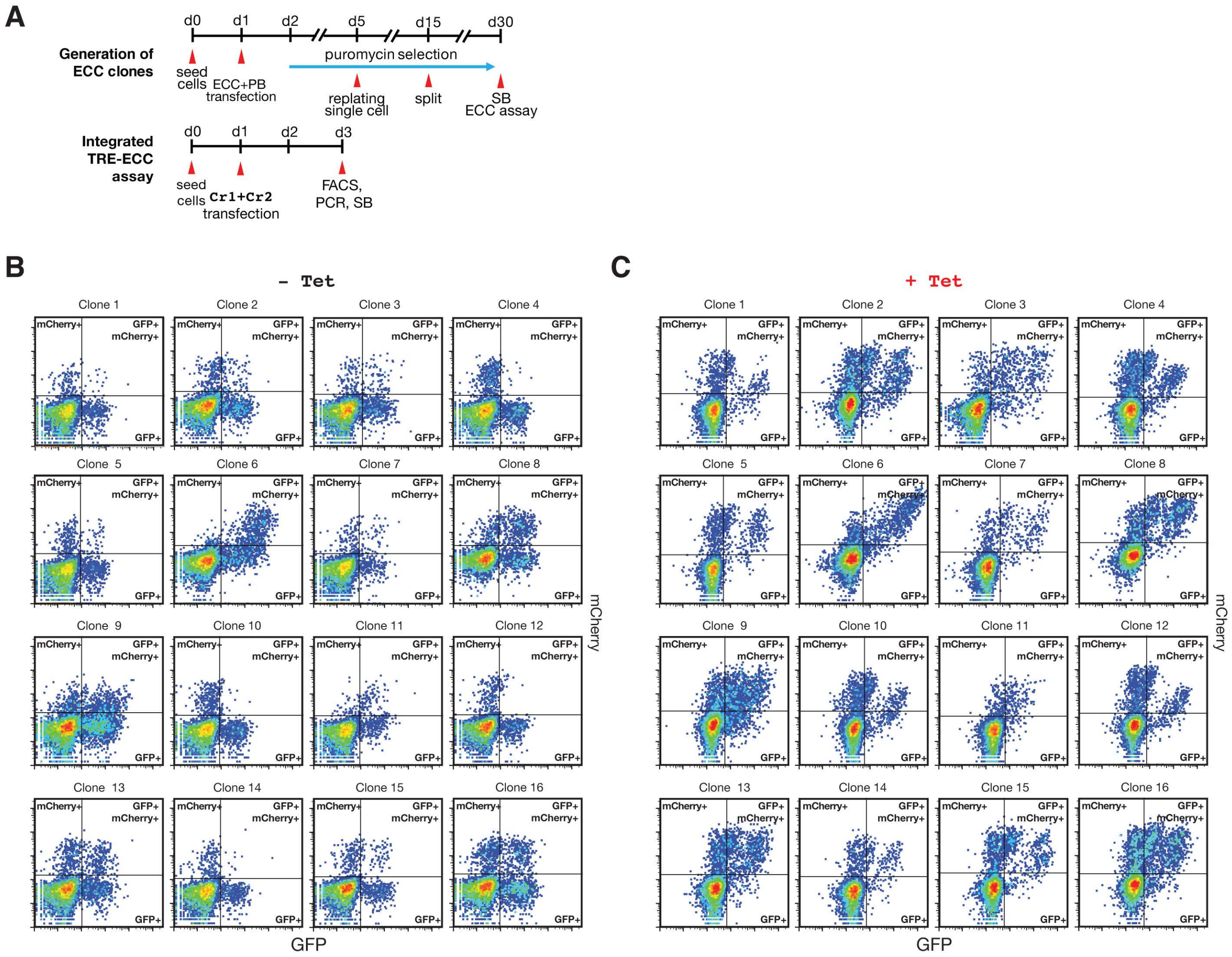
A
Generation of ECC clones
d0
d1
d2
d5
d15
d30
seed cells
ECC+PB transfection
puromycin selection
replating single cell
split
SB ECC assay
Integrated TRE-ECC assay
d0
d1
d2
d3
seed cells
Cr1+Cr2 transfection
FACS, PCR, SB
B
- Tet
C
+ Tet
Clone 1
Clone 2
Clone 3
Clone 4
Clone 5
Clone 6
Clone 7
Clone 8
Clone 9
Clone 10
Clone 11
Clone 12
Clone 13
Clone 14
Clone 15
Clone 16
mCherry+
GFP+ mCherry+
GFP+
mCherry
GFP

Fig. S10

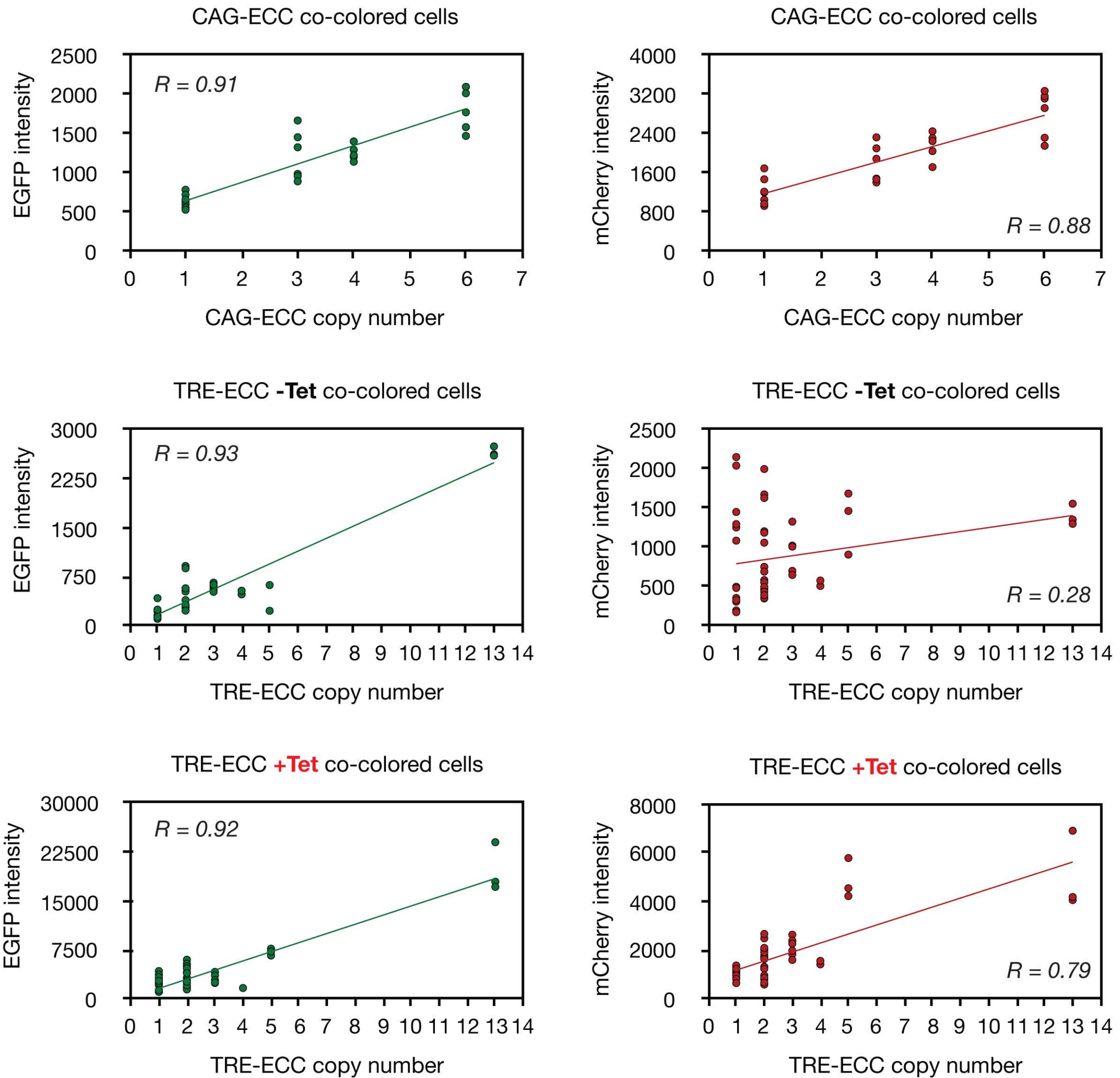
CAG-ECC co-colored cells
EGFP intensity
R = 0.91
CAG-ECC copy number
CAG-ECC co-colored cells
mCherry intensity
R = 0.88
CAG-ECC copy number
TRE-ECC -Tet co-colored cells
EGFP intensity
R = 0.93
TRE-ECC copy number
TRE-ECC -Tet co-colored cells
mCherry intensity
R = 0.28
TRE-ECC copy number
TRE-ECC +Tet co-colored cells
EGFP intensity
R = 0.92
TRE-ECC copy number
TRE-ECC +Tet co-colored cells
mCherry intensity
R = 0.79
TRE-ECC copy number

Fig. S11

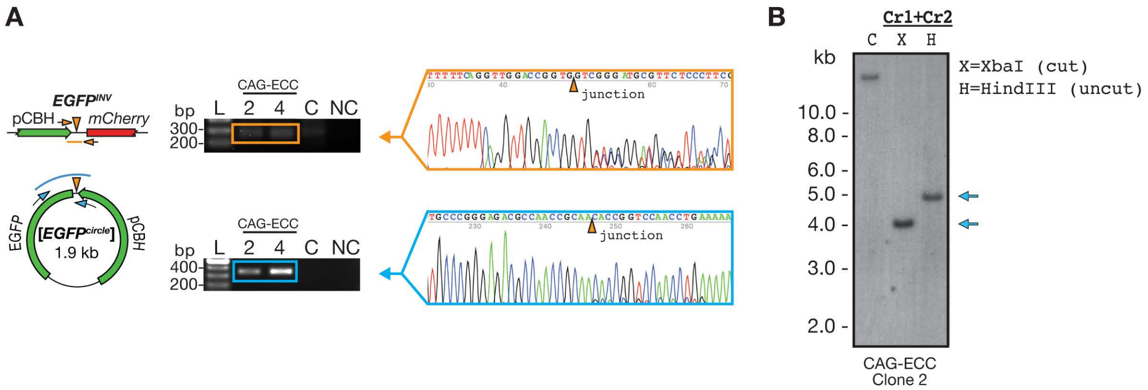
A
EGFP^INV
pCBH
mCherry
CAG-ECC
bp
L
2
4
C
NC
300-
200-
TTTTTCAGGTTGGACCGGTGGTCGGGATGCGTTCTCCCTTCC
junction
[EGFPcircle]
1.9 kb
EGFP
pCBH
400-
200-
TGCCCGGGAGACGCCAACCGCAACACCGGTCCAACCTGAAAAA
junction
B
Cr1+Cr2
C X H
kb
X=XbaI (cut)
H=HindIII (uncut)
10.0 -
8.0 -
6.0 -
5.0 -
4.0 -
3.0 -
2.0 -
CAG-ECC
Clone 2

Fig. S12

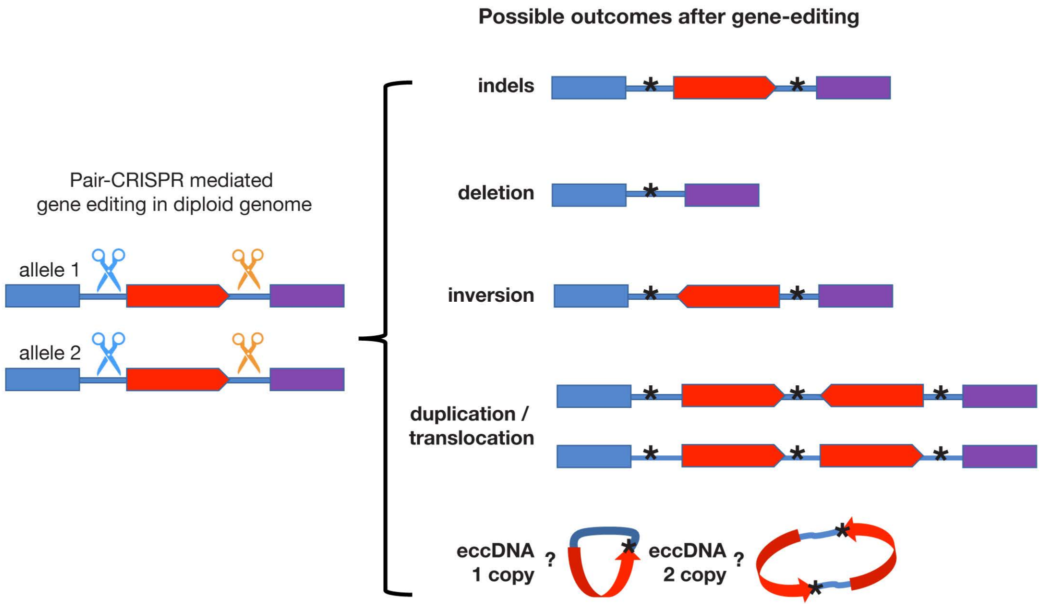
Possible outcomes after gene-editing
Pair-CRISPR mediated
gene editing in diploid genome
allele 1
allele 2
indels
deletion
inversion
duplication /
translocation
eccDNA
1 copy
?
eccDNA
2 copy
?

Fig. S13

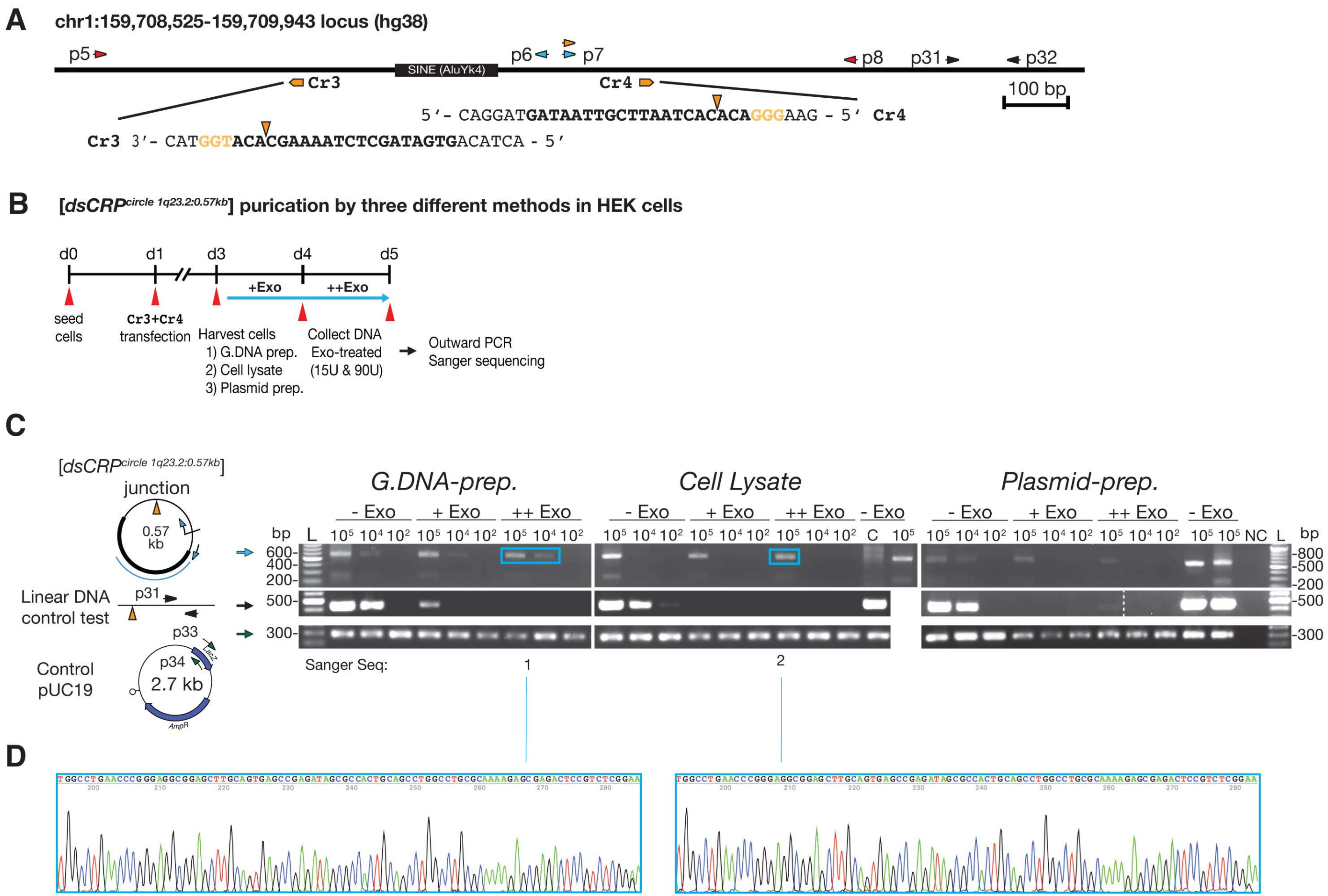

A
chr1:159,708,525-159,709,943 locus (hg38)
p5
p6
p7
p8
p31
p32
SINE (AluYk4)
Cr3
Cr4
100 bp
5'- CAGGATGATAATTGCTTAATCACACAGGGAAG - 5' Cr4
Cr3 3'- CATGGTACACGAAAATCTCGATAGTGACATCA - 5'
B
[dsCRPcircle 1q23.2:0.57kb] purication by three different methods in HEK cells
d0
d1
d3
d4
d5
+Exo
++Exo
seed cells
Cr3+Cr4 transfection
Harvest cells
1) G.DNA prep.
2) Cell lysate
3) Plasmid prep.
Collect DNA
Exo-treated
(15U & 90U)
Outward PCR
Sanger sequencing
C
[dsCRPcircle 1q23.2:0.57kb]
junction
0.57 kb
Linear DNA control test
p31
p33
p34
2.7 kb
Control pUC19
lacZ
AmpR
G.DNA-prep.
Cell Lysate
Plasmid-prep.
- Exo
+ Exo
++ Exo
bp
L
C
NC
600-
400-
200-
500-
300-
-800
-500
-200
-500
-300
Sanger Seq:
1
2
D

Fig. S14

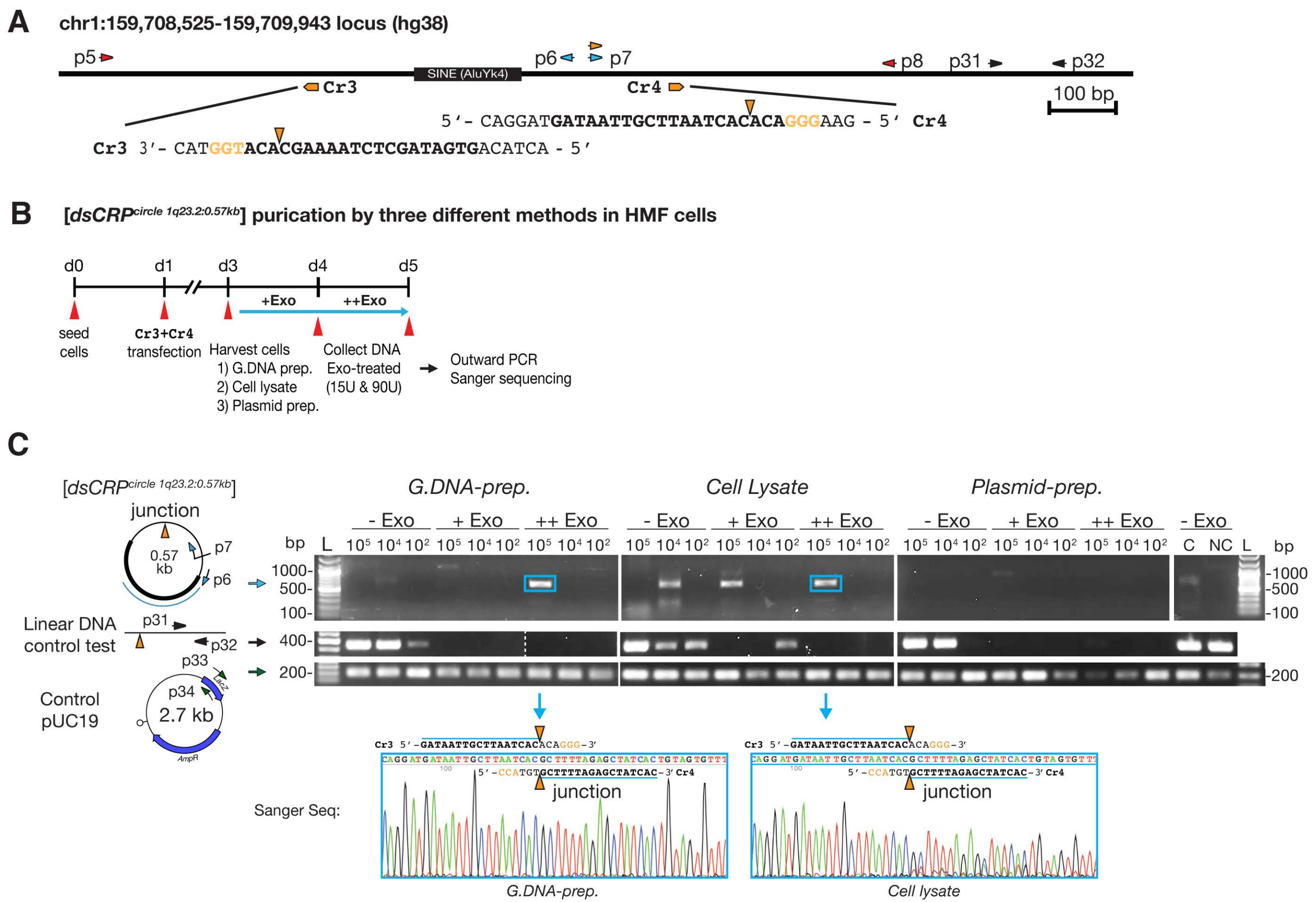
A
chr1:159,708,525-159,709,943 locus (hg38)
p5
SINE (AluYk4)
p6
p7
p8
p31
p32
Cr3
Cr4
100 bp
5'- CAGGATGATAATTGCTTAATCACACAGGGAAG - 5' Cr4
Cr3 3'- CATGGTACACGAAAATCTCGATAGTGACATCA - 5'
B
[dsCRPcircle 1q23.2:0.57kb] purication by three different methods in HMF cells
d0
d1
d3
d4
d5
+Exo
++Exo
seed cells
Cr3+Cr4 transfection
Harvest cells
1) G.DNA prep.
2) Cell lysate
3) Plasmid prep.
Collect DNA
Exo-treated
(15U & 90U)
Outward PCR
Sanger sequencing
C
[dsCRPcircle 1q23.2:0.57kb]
junction
0.57 kb
p7
p6
Linear DNA control test
p31
p32
p33
p34
Control pUC19
2.7 kb
LacZ
AmpR
G.DNA-prep.
Cell Lysate
Plasmid-prep.
- Exo
+ Exo
++ Exo
bp
L
10⁵ 10⁴ 10² 10⁵ 10⁴ 10² 10⁵ 10⁴ 10²
C
NC
1000-
500-
100-
400-
200-
-1000
-500
-100
-200
Sanger Seq:
Cr3 5'-GATAATTGCTTAATCACACAGGG-3'
CAGGATGATAATTGCTTAATCACGCTTTTAGAGCTATCACTGTAGTGTT
100
5'-CCATGTGCTTTTAGAGCTATCAC-3' Cr4
junction
G.DNA-prep.
Cell lysate

Fig. S15

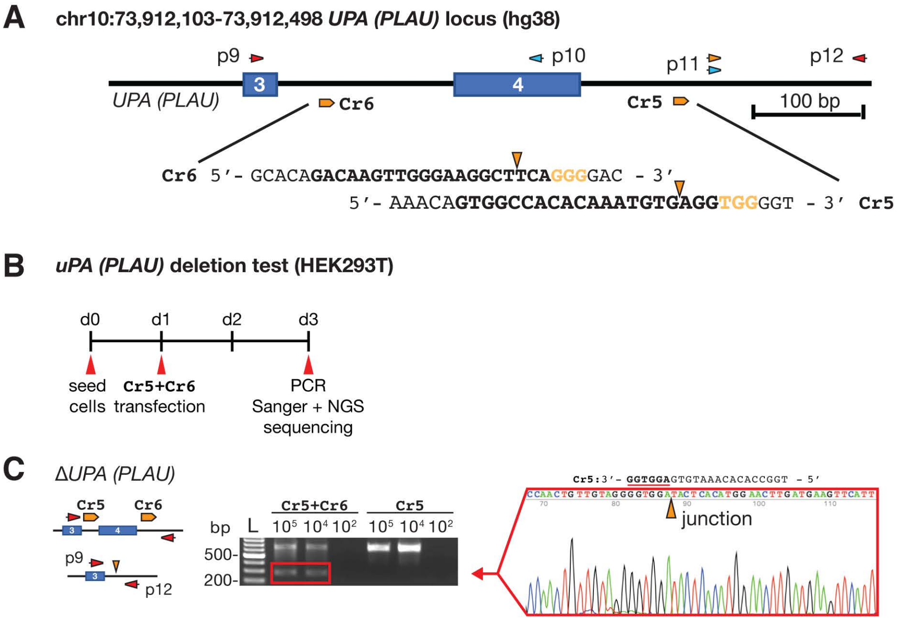
A
chr10:73,912,103-73,912,498 UPA (PLAU) locus (hg38)
p9
p10
p11
p12
3
4
UPA (PLAU)
Cr6
Cr5
100 bp
Cr6 5′- GCACAGACAAGTTGGGAAGGCTTCAGGGGAC - 3′
5′- AAACAGTGGCCACACAAATGTGAGGTGGGGT - 3′ Cr5
B
uPA (PLAU) deletion test (HEK293T)
d0
d1
d2
d3
seed cells
Cr5+Cr6 transfection
PCR
Sanger + NGS sequencing
C
ΔUPA (PLAU)
Cr5
Cr6
3
4
p9
3
p12
Cr5+Cr6
Cr5
bp
L
10⁵ 10⁴ 10²
10⁵ 10⁴ 10²
500-
200-
Cr5:3′- GGTGGAGTGTAAACACACCGGT - 5′
CCAACTGTTGTAGGGGTGGATACTCACATGGAACTTGATGAAGTTCATT
70
80
90
100
110
junction

Fig. S16

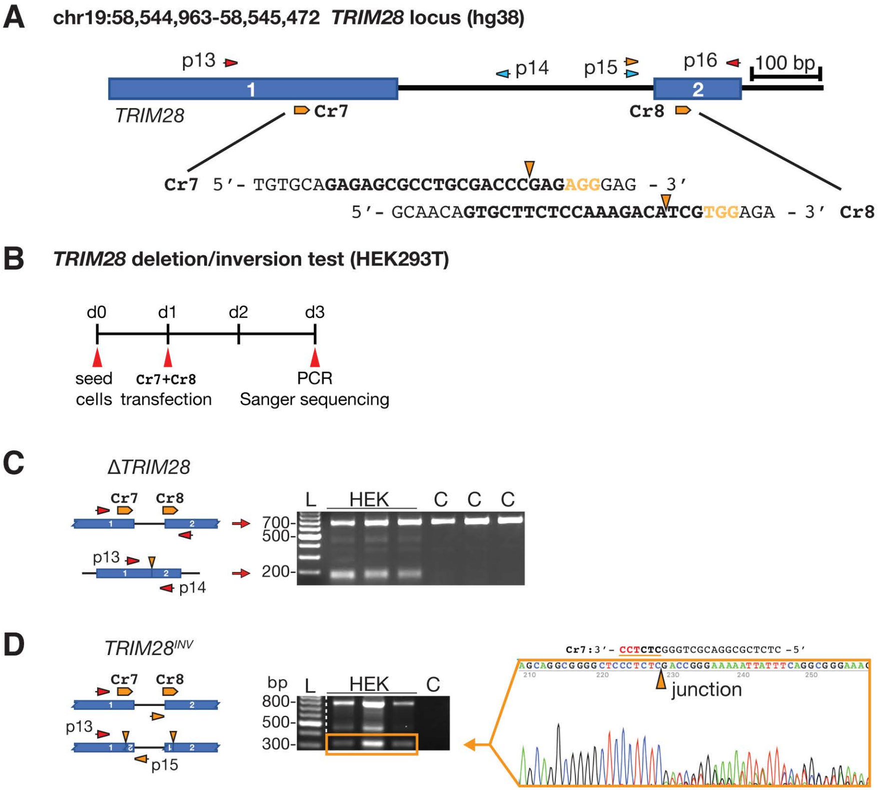
A
chr19:58,544,963-58,545,472 TRIM28 locus (hg38)
p13
p14
p15
p16
100 bp
1
2
TRIM28
Cr7
Cr8
Cr7 5′- TGTGCAGAGAGCGCCTGCGACCCGAGAGGGAG - 3′
5′- GCAACAGTGCTTCTCCAAAGACATCGTGGAGA - 3′ Cr8
B
TRIM28 deletion/inversion test (HEK293T)
d0
d1
d2
d3
seed cells
Cr7+Cr8 transfection
PCR
Sanger sequencing
C
ΔTRIM28
p13
p14
L
HEK
C
700-
500-
200-
D
TRIM28INV
p13
p15
bp
800-
500-
300-
Cr7:3′- CCTCTCGGGTCGCAGGCGCTCTC -5′
AGCAGGCGGGGCTCCCTCTCGACCGGGAAAAATTATTTCAGGCGGGAAA
210
220
230
240
250
junction

Fig. S17

Indels of [*TRIM28*$^{circle\ exon\ 1\text{-}2}$] in HEK cells

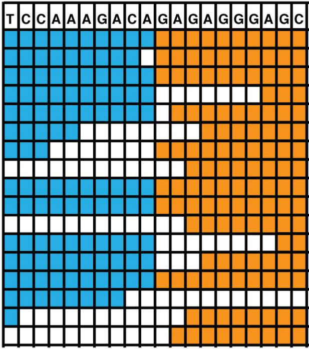

Reads	%	INDEL	Indel Seq. 5'-
24,925	43.27	INS	C
7,579	13.16	DEL	A
3,911	6.79	BEJ*	-
2,901	5.04	DEL	GAGAGGG
2,625	4.56	DEL	G
2,265	3.93	DEL	AGACAGAG
2,182	3.79	DEL&INS	AAAGACA & C
2,101	3.65	DEL	24bp
1,879	3.26	INS	CC
1,690	2.93	INS	TC
1,186	2.06	DEL	12 bp
750	1.30	DEL	GAGAGGGA
540	0.94	DEL	GAG
241	0.42	INS	46 bp
186	0.32	DEL	12 bp
171	0.30	DEL	11bp
!""	0.23	DEL	11bp

Fig. S18

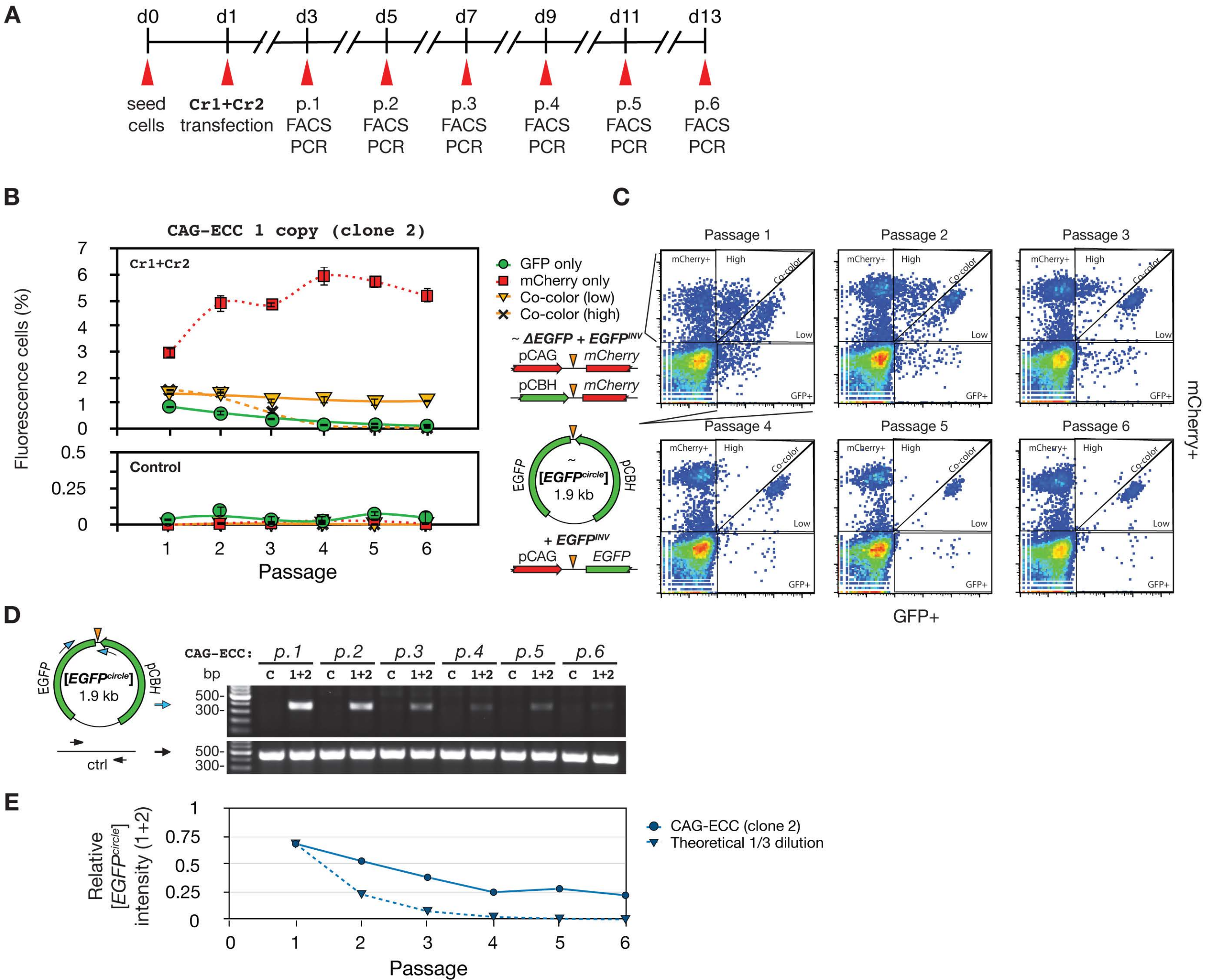
A
d0
d1
d3
d5
d7
d9
d11
d13
seed cells
Cr1+Cr2 transfection
p.1 FACS PCR
p.2 FACS PCR
p.3 FACS PCR
p.4 FACS PCR
p.5 FACS PCR
p.6 FACS PCR
B
CAG-ECC 1 copy (clone 2)
Cr1+Cr2
Control
Fluorescence cells (%)
Passage
GFP only
mCherry only
Co-color (low)
Co-color (high)
~ ΔEGFP + EGFP^INV
pCAG
mCherry
pCBH
mCherry
~ [EGFP^circle] 1.9 kb
EGFP
pCBH
+ EGFP^INV
pCAG
EGFP
C
Passage 1
Passage 2
Passage 3
Passage 4
Passage 5
Passage 6
mCherry+
High
Co-color
Low
GFP+
D
[EGFP^circle] 1.9 kb
EGFP
pCBH
ctrl
CAG-ECC:
p.1
p.2
p.3
p.4
p.5
p.6
bp
C
1+2
500-
300-
E
Relative [EGFP^circle] intensity (1+2)
Passage
CAG-ECC (clone 2)
Theoretical 1/3 dilution

Fig. S19

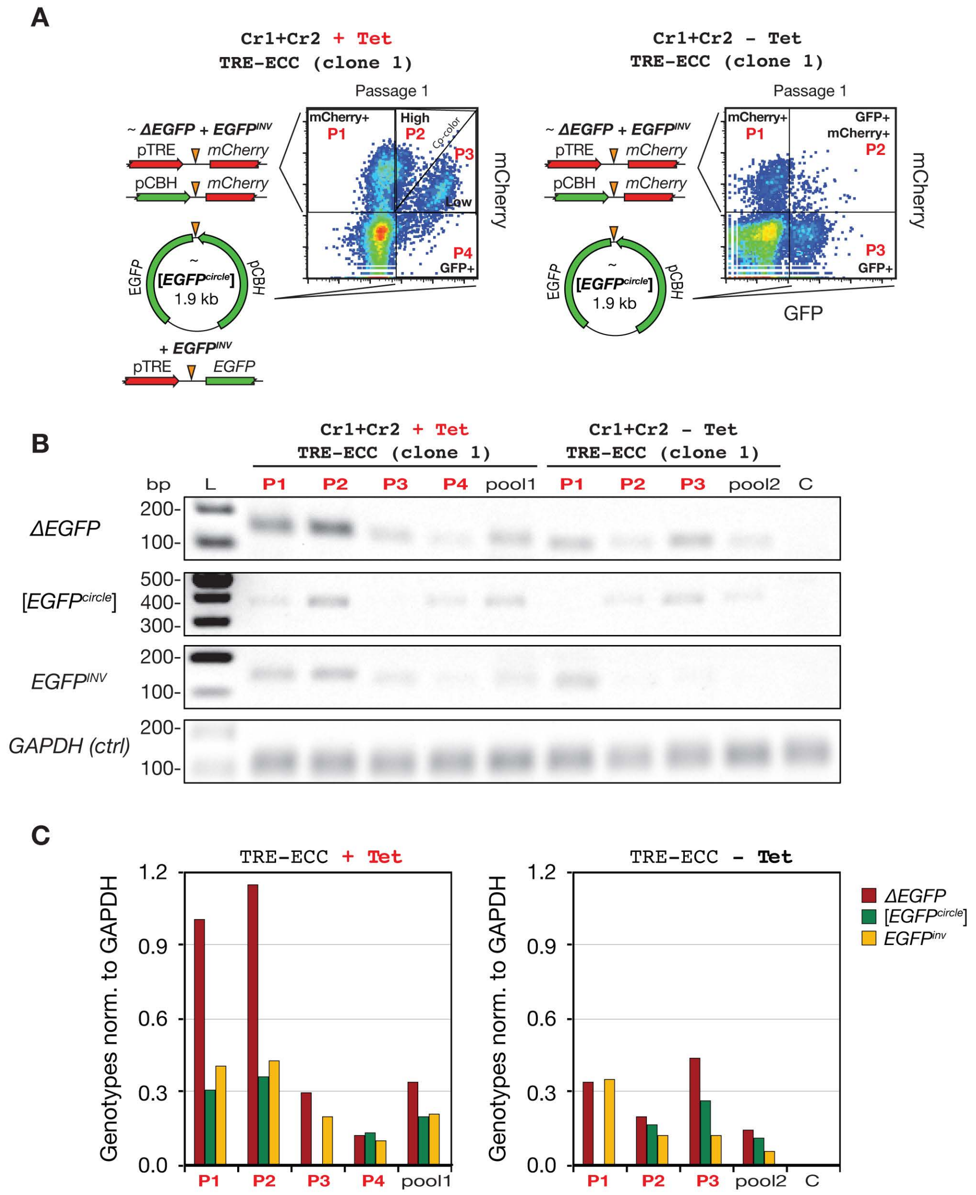
A
Cr1+Cr2 + Tet
TRE-ECC (clone 1)
Passage 1
~ ΔEGFP + EGFP^INV
pTRE
mCherry
pCBH
mCherry
EGFP
[EGFP^circle]
1.9 kb
pCBH
+ EGFP^INV
pTRE
EGFP
mCherry+
P1
High
P2
Co-color
P3
Low
P4
GFP+
mCherry
Cr1+Cr2 - Tet
TRE-ECC (clone 1)
Passage 1
mCherry+
P1
GFP+
mCherry+
P2
P3
GFP+
GFP
B
Cr1+Cr2 + Tet
TRE-ECC (clone 1)
Cr1+Cr2 - Tet
TRE-ECC (clone 1)
bp
L
P1
P2
P3
P4
pool1
P1
P2
P3
pool2
C
ΔEGFP
200-
100-
[EGFP^circle]
500-
400-
300-
EGFP^INV
200-
100-
GAPDH (ctrl)
200-
100-
C
TRE-ECC + Tet
TRE-ECC - Tet
Genotypes norm. to GAPDH
1.2
0.9
0.6
0.3
0.0
P1
P2
P3
P4
pool1
P1
P2
P3
pool2
C
ΔEGFP
[EGFP^circle]
EGFP^inv

Fig. S20

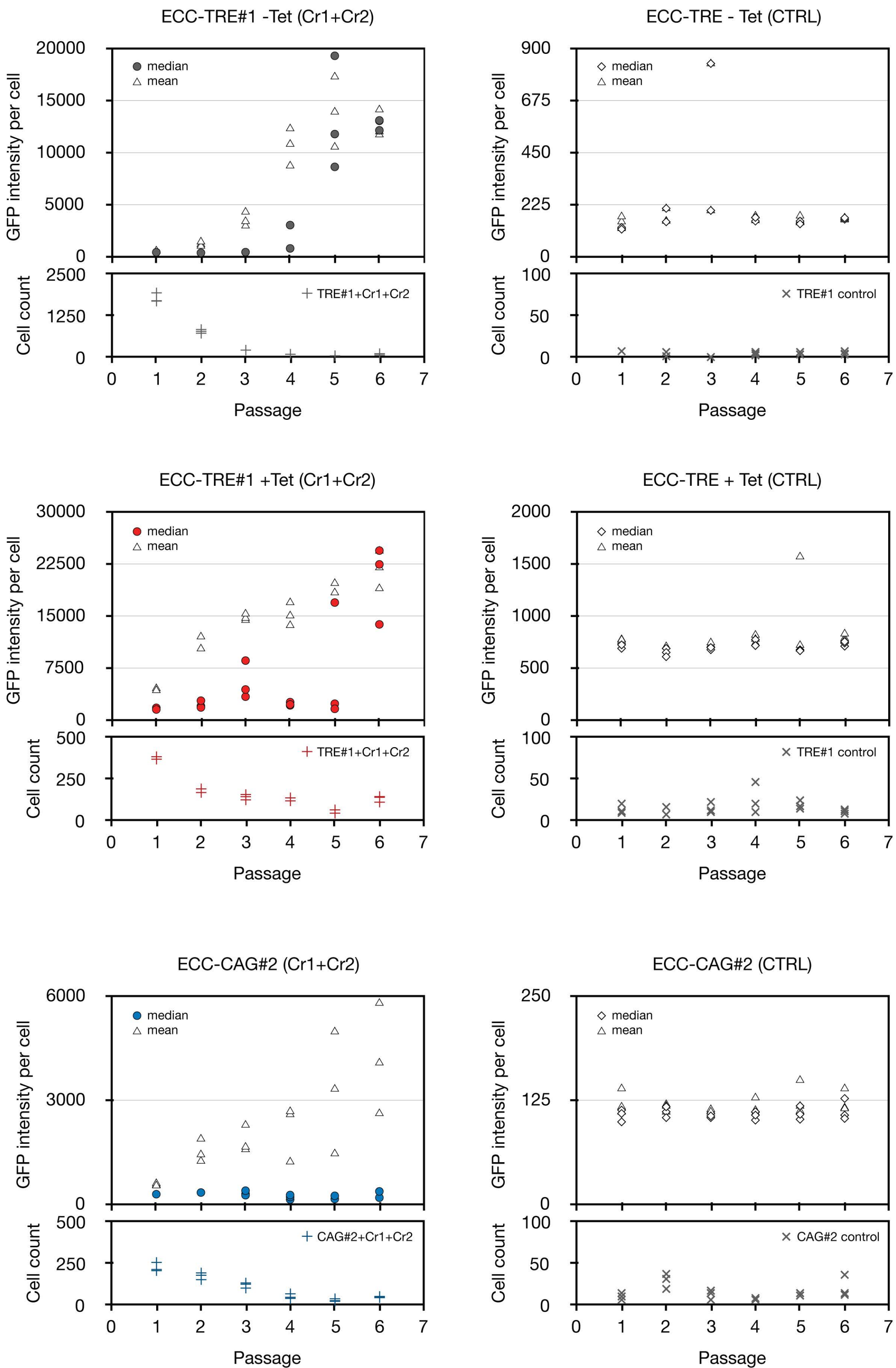
ECC-TRE#1 -Tet (Cr1+Cr2)
ECC-TRE - Tet (CTRL)
ECC-TRE#1 +Tet (Cr1+Cr2)
ECC-TRE + Tet (CTRL)
ECC-CAG#2 (Cr1+Cr2)
ECC-CAG#2 (CTRL)
median
mean
GFP intensity per cell
Cell count
Passage
TRE#1+Cr1+Cr2
TRE#1 control
CAG#2+Cr1+Cr2
CAG#2 control

Fig. S21

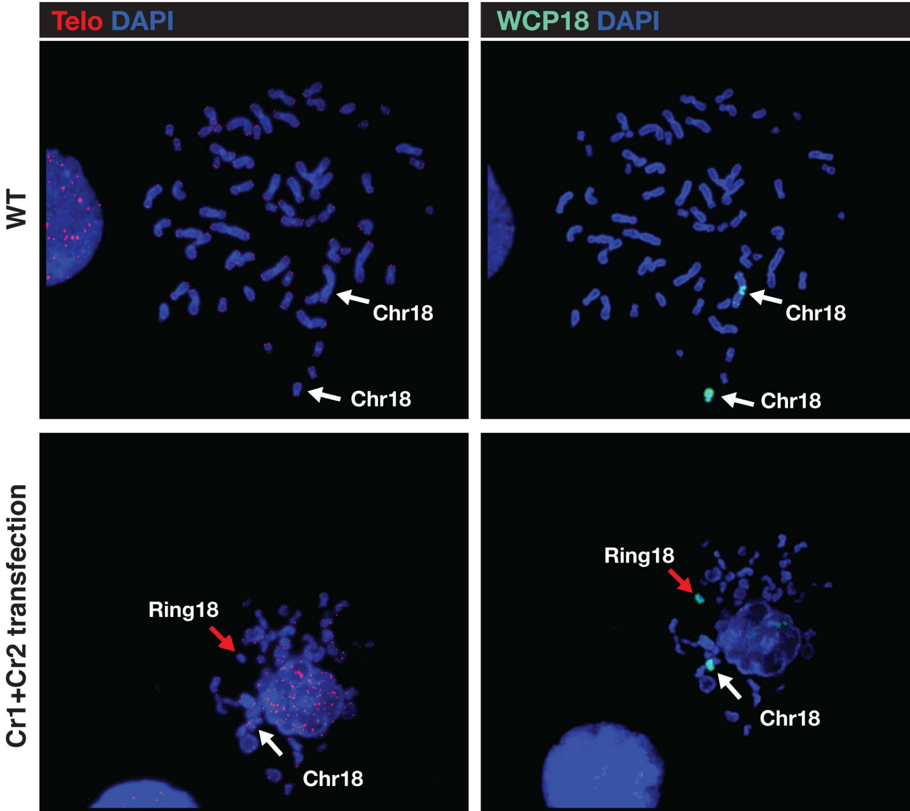
Telo DAPI
WCP18 DAPI
WT
Cr1+Cr2 transfection
Chr18
Chr18
Chr18
Chr18
Ring18
Chr18
Ring18
Chr18
